# Unified feature association networks through integration of transcriptomic and proteomic data

**DOI:** 10.1371/journal.pcbi.1007241

**Published:** 2019-09-17

**Authors:** Ryan S. McClure, Jason P. Wendler, Joshua N. Adkins, Jesica Swanstrom, Ralph Baric, Brooke L. Deatherage Kaiser, Kristie L. Oxford, Katrina M. Waters, Jason E. McDermott

**Affiliations:** 1 Biological Sciences Division, Pacific Northwest National Laboratory, Richland WA, United States of America; 2 Department of Microbiology and Immunology, School of Medicine, University of North Carolina, Chapel Hill, Chapel Hill, NC, United States of America; 3 Signatures Science and Technology Division, Pacific Northwest National Laboratory, Richland WA, United States of America; 4 Department of Molecular Microbiology and Immunology, Oregon Health & Sciences University, Portland, OR, United States of America; University of Illinois at Urbana-Champaign, UNITED STATES

## Abstract

High-throughput multi-omics studies and corresponding network analyses of multi-omic data have rapidly expanded their impact over the last 10 years. As biological features of different types (e.g. transcripts, proteins, metabolites) interact within cellular systems, the greatest amount of knowledge can be gained from networks that incorporate multiple types of -omic data. However, biological and technical sources of variation diminish the ability to detect cross-type associations, yielding networks dominated by communities comprised of nodes of the same type. We describe here network building methods that can maximize edges between nodes of different data types leading to integrated networks, networks that have a large number of edges that link nodes of different–omic types (transcripts, proteins, lipids etc). We systematically rank several network inference methods and demonstrate that, in many cases, using a random forest method, GENIE3, produces the most integrated networks. This increase in integration does not come at the cost of accuracy as GENIE3 produces networks of approximately the same quality as the other network inference methods tested here. Using GENIE3, we also infer networks representing antibody-mediated Dengue virus cell invasion and receptor-mediated Dengue virus invasion. A number of functional pathways showed centrality differences between the two networks including genes responding to both GM-CSF and IL-4, which had a higher centrality value in an antibody-mediated vs. receptor-mediated Dengue network. Because a biological system involves the interplay of many different types of molecules, incorporating multiple data types into networks will improve their use as models of biological systems. The methods explored here are some of the first to specifically highlight and address the challenges associated with how such multi-omic networks can be assembled and how the greatest number of interactions can be inferred from different data types. The resulting networks can lead to the discovery of new host response patterns and interactions during viral infection, generate new hypotheses of pathogenic mechanisms and confirm mechanisms of disease.

## Author summary

Inferring co-expression networks that combine multiple types of–omics data can lead to networks that are highly segregated with very few edges linking nodes of different–omics types. Here, we explored methods of improving integration in co-expression networks and found that a random forest approach, GENIE3, was, in many cases, the best method for inferring such networks. GENIE3 was approximately as accurate as other network inference methods so this improvement in integration did not come at the cost of network quality. We also used GENIE3 to infer several networks using data derived from infection of human cells with Dengue virus and interrogated them to better understand processes that may have higher importance when Dengue virus carries out antibody-mediated infection compared to receptor-mediated infection. Processes involved in GM-CSF and IL-4 were more central during antibody-mediated infection. Overall, these studies identify network inference methods that are particularly well suited to multi-omic networks and also demonstrate how these approaches can be used to better understand Dengue virus infection.

## Introduction

Over the last several decades, a number of multi-omic experimental methods have emerged to study host-pathogen interactions [1] and other complex biological systems. These studies focus on collecting a particular type of molecule from a biological system and using high throughput methods to query abundance levels across changing conditions. These empirical methods include transcriptomics [2–5], proteomics [6–8], metabolomics [9, 10], and lipidomics [11–13]. Host-pathogen interactions, including immunological responses and virulence factors, represent a particularly complex biological site where multiple different types of biomolecules are known to play important roles. Therefore, some of the most critical insights and translational conclusions can be gained from combining different types of -omics-based studies. Kocharunchitt et. al. examined the response of *Escherichia coli* O157:H7 to varying water temperatures, emulating host interactions, by collecting proteomic and transcriptomic data and using both to query responses of a single parent gene [14]. Dapat and Oshitani linked host and viral proteins of respiratory syncytial virus based on creating networks of protein-protein interactions from pre-existing databases and then overlaying abundance data from transcriptomic and proteomic studies onto these networks to identify hubs that may alter their expression [15].

Other studies have used transcriptomics, proteomics, and lipidomics in integrated networks to elucidate interactions between Hepatitis C virus and the host [16, 17] or to better understand bacterial virulence programs [18, 19]. Several multi-omic studies have reported on the fact that, globally, there seems to be poor correlation between different data types (transcriptomic or proteomic for example) that are derived from the same gene [20–22]. Although expression heterogeneity (batch effects) can be an important driver of poor cross-class correlation, this lack of correlation is also likely due to the regulatory processes that determine abundance of each transcript and protein (e.g. transcription, translation, degradation, etc.). Lack of correlation may also be linked to the inherent differences in querying protein levels (2D gel electrophoresis and mass spectrometry) compared to transcript levels (microarray or RNA-seq) or in other aspects of experimental design [23].

Because–omics data tends to be large and complex a network approach that links features in omics datasets can be very useful in gaining a high level view of the system and identifying which features occupy positions of high centrality or which processes are co-regulated in a non-intuitive way. A number of methods exist for inferring networks based on correlation coefficient, mutual information, Bayesian probability, random forest analysis and regression analysis. Networks of related features have been made for transcriptomic analyses of pathogens [24, 25], for proteome analyses [17, 26] and for metabolomic or lipidomic analyses [27]. Such networks have been used to identify specific processes or pathways that may be responding in tandem across a range of conditions, providing insight into coordination and cross-talk in biological systems as they respond to infection. They can also be used to identify genes of high importance to the organism under analysis [24, 28, 29], predict gene function [30–32] or identify regulatory strategies of biological systems [33–35]. Networks focusing on pathogens have identified which genes may be specifically important to virulence [24, 36] and other networks have been focused on expanding this approach further by querying not only the host or pathogen but specifically interactions between these organisms [37–39].

As networks seek to model complex biological systems with a number of different molecular features (e.g. transcripts, proteins, metabolites), the most accurate networks will be those that can incorporate multiple types of–omics data reporting on these molecules. The lack of apparent correlation between different–omics types (protein and transcript) described above has also emerged when networks of multiple data types are constructed. Despite this hurdle, approaches that effectively integrate data across different technological platforms and biomolecular classes are likely to become more important as multi-omics data becomes easier to generate. Platforms such as mass-spectrometry and RNA-seq also result in missing values when molecules fall below an abundance threshold, and attempts have been made to develop methodologies to incorporate these data types when building networks using mutual information scores [40, 41]. With the goal of creating improved integrated networks, we examine here a number of network inference tools using several types of–omics data (mainly transcriptomic and proteomic) and identify those inference tools that create the most integrated networks, defined as those having the maximum number of edges connecting different types of–omics data, termed cross-type edges. Previous studies have focused on ranking network inference methods based on accuracy and have found that GENIE3, a random forest method, created the best network in terms of its ability to link known regulator-target pairs in *Escherichia coli* [42]. However, there has been no corresponding analysis that systematically ranks network inference methods by their ability to create integrated networks of transcripts and proteins. We perform this ranking here and find that, in most cases, GENIE3 is also the best inference method to create integrated networks of proteomic and transcriptomic data. We show that these networks, including the cross-type edges in the network, are accurate, and we use this approach to interrogate and compare networks inferred from data derived from antibody-mediated entry of Dengue virus into cells and from receptor-mediated entry. The methods presented here provide important guidance for constructing multi-omic networks representing host responses to infection, and offer strategies for inferring networks that can act as highly accurate and robust models of cellular systems.

## Results

### Networks of transcripts and proteins are highly segregated

**Fig 1:**
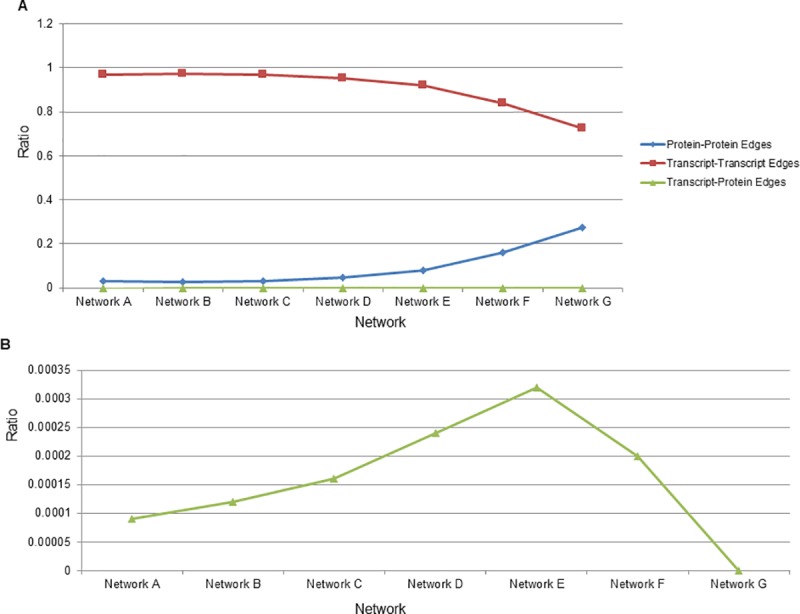
Ratio of edges types by Pearson correlation coefficient to all existing edges of that type. (A) The network letter is indicated on x-axis and the ratio of the number of edges of a particular type (either Transcript-Transcript, Protein-Protein, or Transcript-Protein) to the total number of edges of all types is shown on the y-axis. Network A is the largest and network G the smallest. Protein-Protein ratios are represented by blue diamonds, Transcript-Transcript ratios are represented by red squares and Cross-type edges (Transcript-Protein) are represented by green triangles. (B) Cross-type edge ratios are enlarged for ease of viewing. Network G inferred with Pearson had no cross-type edges so the resulting ratio is zero.

Transcriptomic and proteomic samples were collected from cells at 2, 8, 16, and 24 hours post-infection. We initially carried out functional enrichment of transcripts and proteins that showed statistically significant changes in expression as a function of infection. This showed that transcripts showing changes in expression in response to infection were enriched for processes such as cytokine signaling, TLR signaling, phagosomes, viral carcinogenesis and response to infection (S1 and S2 Tables). Proteins showing changes in expression as a function of infection were enriched for some of the same pathways as well as regulation of the cytoskeleton and antigen presentation (S3 Table). We also examined replicate and treatment variation between samples as part of a quality analysis of the Dengue dataset. Using an Adonis test we found that Treatments (virus infection, cell type, etc.) accounted for 23.6% of the differences between samples when proteins were examined and 40.7% of the differences when transcripts were examined with both of these results being statistically significant (*p*-value > 0.001 for both tests). In contrast, replicates accounted for < 3% of differences among samples when either proteins or transcripts were examined with neither of these results being significant (*p*-value of 0.858 and 0.981 respectively). Networks were then inferred for this dataset where transcripts and proteins were kept as separate nodes in the network. The same gene detected at the transcript or protein level is represented by two separate nodes in the network (though often a gene is only detected at either transcript or protein level, not both). This was done for two reasons: (1) a gene may show different expression levels as a transcript or as a protein due to post-transcriptional regulation. Combining transcript and protein levels may therefore give an inaccurate overall view of the gene’s expression level. (2) Within the cell transcripts and proteins exist simultaneously and may interact with each other and affect each other’s expression. To identify these putative interactions it will be necessary to also keep transcript and proteins as separate entities in a network. Initial networks using both proteins and transcripts as separate features in the network and Pearson correlation coefficient (PCC) as an association method resulted in an extremely low number of edges linking transcripts and proteins compared to those linking transcripts only or proteins only (**S1 Fig, S4 Table**). Using a PCC threshold of ~0.85 produced a network containing 100000 total edges and 5016 total nodes. Within this network, 97,396 edges (97.3%) connected two transcripts, 2592 (2.59%) connected two proteins and only 12 (0.012%) connected a protein and transcript (cross-type edges). This low number of cross-type edges persisted as additional Pearson thresholds examined, ranging from ~0.81 to ~0.96 and producing networks of 200000, 50000, 25000, 12500, 5000 and 2500 edges (**Fig 1A and 1B**). We next examined whether the differing scales and distributions of proteomic vs. transcriptomic data could be the cause of the lack of cross-type edges. Because of differences in the nature of microarrays compared to mass spectrometry and the downstream data analysis expression values for transcripts are generally far lower than expression values for proteins (**S2A Fig**). To artificially correct for this discrepancy, we multiplied all transcriptomic gene expression values by a factor of 2.6. This led to modified transcriptomic values that had a distribution and scale that was highly similar that seen with proteomic expression values (**S2B Fig**). However, when using these modified transcriptomic values, we found that there was no change in the number of cross-type edges inferred (**S4 Table**). This demonstrates that the differing scales and distributions of transcriptomic vs. proteomic data seen here are not the cause of the lack of cross-type edges and that other factors are at play.

**Fig 2:**
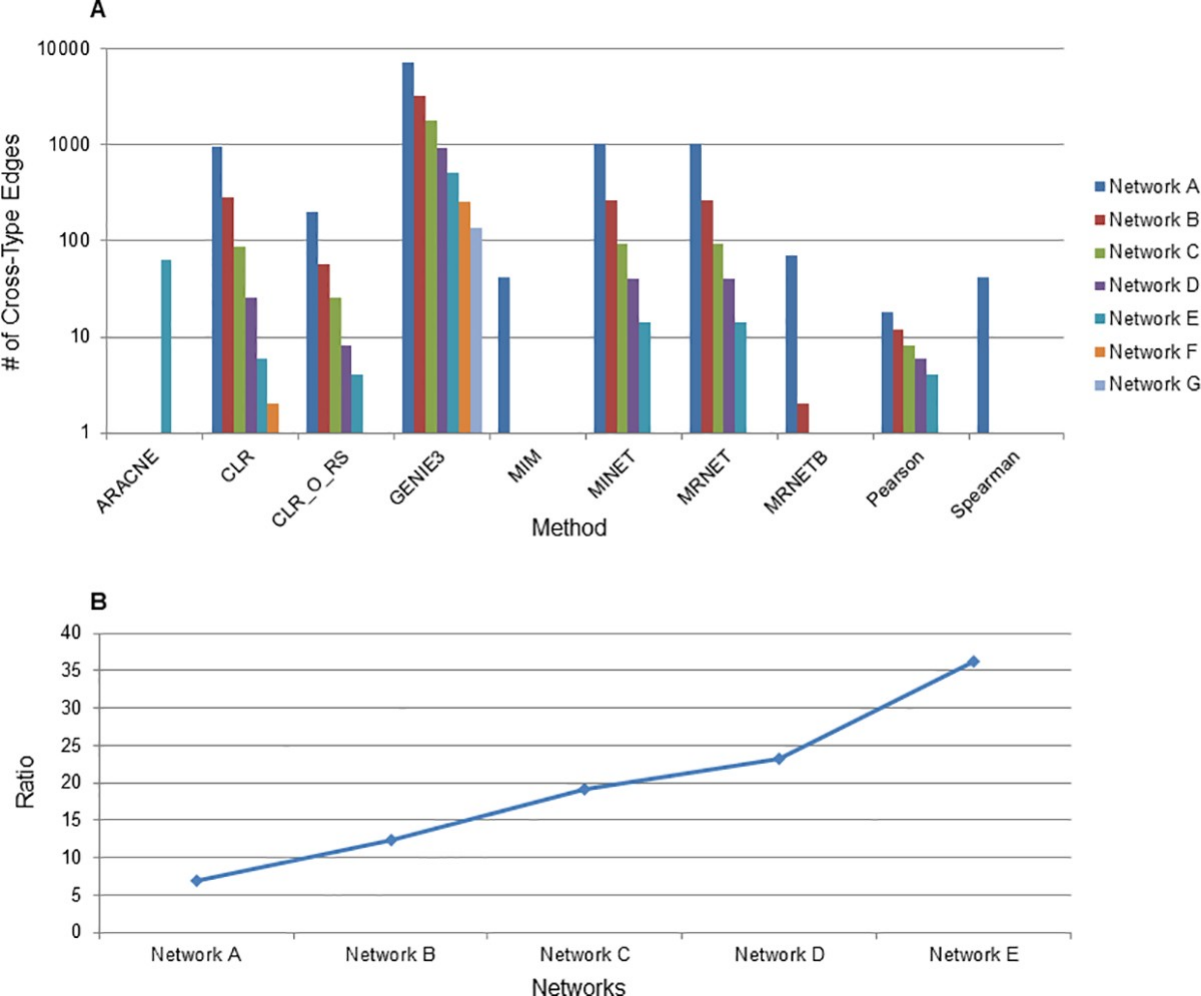
Comparison of network inference method’s ability to generate cross-type edges. (A) Networks were made with several different methods and edge cutoffs were chosen so that all networks of a particular letter are the same size across inference methods. The number of Cross-type edges for each network is shown on the y-axis and the methods on the x-axis. CLR_O_RS indicates the original CLR algorithm with resampling to distinguish it from the version of CLR used in the MINET package. The ARACNE method removes edges of low association as part of its methodology and thus only smaller networks containing high scoring edges are retained with ARACNE. (B) The fold increase in Cross-type edges inferred by the GENIE3 method compared to the MINET method (the next best method at creating Cross-type edges) is shown. For networks F and G MINET did not draw any Cross-type edges and so these ratios are not displayed.

There are biological functions that may cause proteins and transcripts to cluster together [22], but it is also likely that the inference method of choice contributes to the segregation of the network. To explore this further we investigated other methods that may be able to create more integrated networks. Aside from PCC, other methods that can be used to link features include Spearman correlation coefficient, and those developed to infer transcriptional regulatory interactions from transcriptomic data, but are applicable to inference of associations between different data types, such as mutual information [33, 43] and random forest methods. We examined nine other network inference algorithms in addition to PCC to assess their ability to infer cross-type edges. These 10 inference methods are described in the Methods and include correlation coefficient (Pearson and Spearman), mutual information (CLR, methods from the MINET package) and random forest (GENIE3) approaches. We specifically were interested in GENIE3 as it ranked as the top performer in the DREAM5 challenge and had been used in the past to infer networks of proteins and transcripts [23]. For each method we inferred seven different networks ranging in size from 200000 to 2500 edges to evaluate the performance of methods. Networks with the same letter designation (Networks A through G) are matched by size across inference methods. Network designations, the edge cutoffs used, the sizes of networks and the number of cross-type edges are shown in **S4 Table** and .sif files for each network are included as a Supplementary Dataset. While a number of the mutual information based methods improved upon PCC in drawing cross-type edges, GENIE3, the random forest method, was by far the best method for creating integrated networks (**Fig 2A**). It was 6.9-fold better than the next best method (MINET, from the MINET package in R) when examining the largest networks. When examining small networks of 5000 edges it produced 252 cross-type edges, compared to only two for CLR, one for PCC and zero for all other methods (**S3 Fig, S4 Table**). Similar to PCC, increasing the threshold used to define an edge to make it more stringent led to an increase in the ratio of cross-type edges to total edges. We also found that increasing the edge threshold led to an increase in the ratio of cross-type edges drawn by GENIE3 compared to those drawn by MINET (**Fig 2B**), at smaller network sizes GENIE3 has an even larger advantage over other network inference methods.

### Jaccard similarity of network inference methods

**Fig 3:**
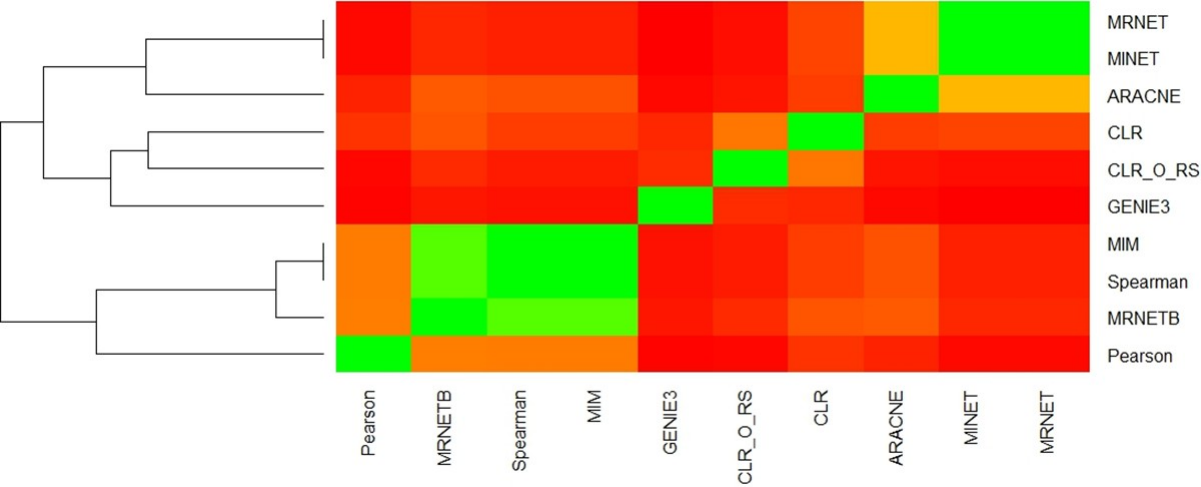
Comparisons of edge identity in networks. Overlap of identical edges is shown for all network inference methods by calculating the Jaccard similarity of edges (the ratio of intersection of edges/union of edges) when comparing two networks. Green indicates higher similarity and red lower similarity. Inference methods were clustered and the resulting dendogram is shown on the left. CLR_O_RS indicates the original CLR algorithm with resampling to distinguish it from the version of CLR used in the MINET package.

As there were large differences in the ability of each method to infer cross-type edges, we next compared how much edge overlap there was between methods. Using networks of 5000 edges from each method we determined the overlap of identical edges between networks. Networks were compared using the Jaccard similarity of edges (the ratio of intersection of edges/union of edges). **Fig 3** shows the similarity of all network pairs of the 10 inference methods we tested, with green representing higher Jaccard similarity and red lower similarity. The composition of the GENIE3 network was closest to networks made using CLR from the MINET package and the original CLR algorithm using resampling (Methods). The Jaccard similarity of edges comparing GENIE3 to these methods was 0.174 and 0.184 respectively. When examining the pattern of overlap between other algorithm pairs, two additional clusters emerge. The MRNET, MINET, and ARACNE methods from the MINET package cluster together showing Jaccard similarity of approximately 0.42. Clustering with PCC is the Spearman correlation coefficient and two other methods from the MINET package, MIM and MRNETB, the average Jaccard similarity between these methods being 0.612. There are several clusters of methods that produce similar networks, but GENIE3 produces networks that have more unique edges distinct from networks produced by the other methods. Therefore, GENIE3 is able to detect cross-platform relationships that are not detected by other methods.

### Quality of network edges

**Fig 4:**
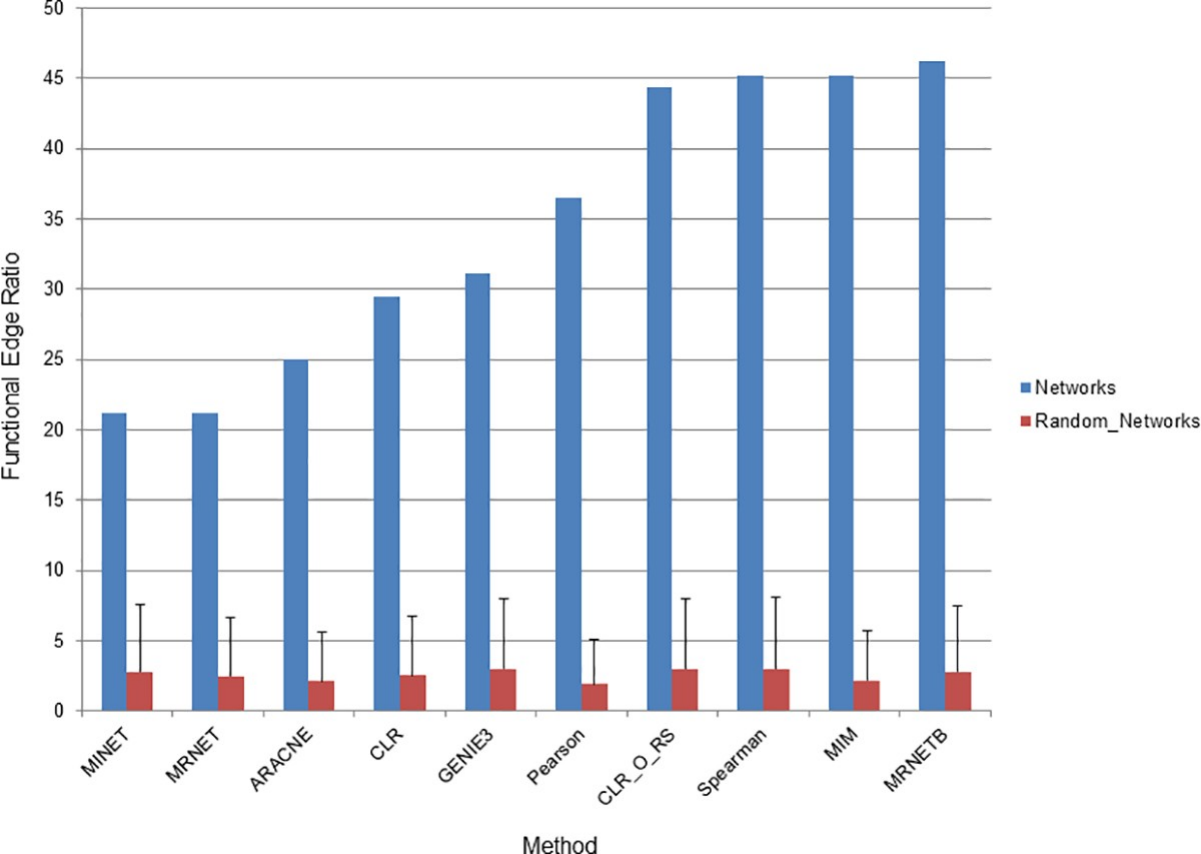
Quality of network inference methods. The ability of networks to connect features in the same functional category was interrogated. The ratio of edges (as a percentage) connecting annotated features in the same functional category to all edges connecting annotated features is displayed on the y-axis. The network inference method is displayed on the x-axis. A network of 5000 edges was examined for each network inference method. Blue bars represent ratios of edges in the networks described here and red bars represent ratios of edges when all nodes in the network have been randomized. Error bars indicate standard deviation of ratios from three randomized networks. CLR_O_RS indicates the original CLR algorithm with resampling to distinguish it from the version of CLR used in the MINET package.

Because there is some variation in overlap of edges between methods (ratios range from 0.104 to 0.99), we next wanted to examine how accurate networks were to address the question, “are different network inference methods producing edges of different quality?” Quality of networks can be difficult to assess as they generally provide far more information than has been experimentally verified. Previous studies have graded networks based on their ability to link known regulator-target pairs in *E*. *coli* and in this study GENIE3 emerged as the most accurate [42]. However, networks provide additional information beyond regulator-target pairs and we chose a wider metric based on linking genes in the same functional group. Here, we focus on the number of edges that connect two features that are in the same functional group as determined using KEGG pathways [44, 45]. Because there is such a large difference in the ability of each inference method to infer cross-type edges, we used only transcript-transcript edges for this analysis. This approach revealed that there was moderate variability between network inference methods (**Fig 4**). MRNETB had the highest quality network according to this metric with a functional edge ratio of 46.1%. MINET showed the lowest functional edge ratio at 21.2% while GENIE3 was near the middle with a functional edge ratio of 31.1%. As a baseline we also examined the quality of networks where the identity of all nodes had been randomized. All of the networks improved upon the randomization, which had a functional edge ratio clustered around 7.6 ± 0.012%. Importantly, the method we identified as producing the greatest number of cross-type edges, GENIE3, produced edges of approximately the same quality as the other methods. This indicates that the significant improvement of GENIE3 in inferring cross-type edges does not come at the expense of network quality. This is consistent with prior studies using GENIE3 on bacterial systems, which have also found it to be highly accurate [42].

**Fig 5:**
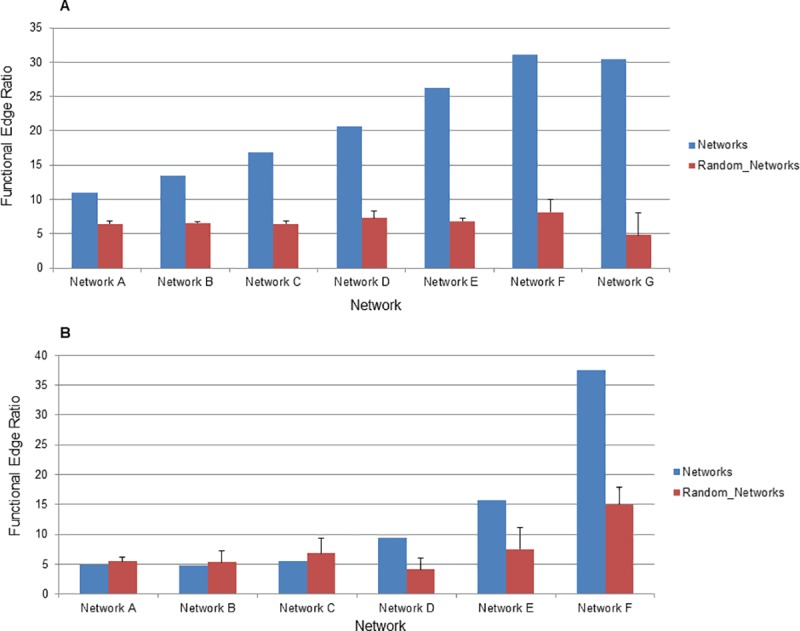
Quality of GENIE3 across increasing edge cutoffs. (A) The ratio of Transcript-Transcript edges (as a percentage) connecting annotated features in the same functional category to all edges connecting annotated features is displayed on the y-axis. The network is displayed on the x-axis. Blue bars represent ratios of edges in the networks described here and red bars represent ratios of edges in randomized networks. Error bars indicate standard deviation of ratios from three randomized networks. (B) The same ratio is shown for Cross-type edges specifically.

Having shown that the large increase in cross-type edges produced by GENIE3 does not come at the expense of network quality, we next examined how the edge threshold affects the quality of the networks produced by GENIE3 as assessed by functional edge ratio. Increasing the edge threshold leads to networks of higher quality, although even at the least stringent threshold used in this study the resulting 200000 edge network is still better than a randomized network of the same size, showing that networks can be extremely large and still contain high quality information (**Fig 5A**). It should also be noted that the quality of networks followed an exponential curve as network size was decreased; networks rapidly increase in quality at sizes of 50000 edges or less. The analysis in **Fig 5** examines only transcript-transcript edges, however, the specific advantage of GENIE3 is its ability to infer cross-type edges. Cross-type edges are far sparser and also are likely to be affected by biological aspects that limit correlation between transcripts and proteins such as post-transcriptional regulation and differences in experimental platform. We next wanted to confirm that cross-type edges, despite being more difficult to infer, also convey high quality functional information. We therefore determined the functional edge ratio of cross-type edges specifically in networks inferred using GENIE3. This analysis showed that cross-type edges made by GENIE3 are of lower functional quality than transcript-transcript edges. However, they are still better than edges produced in randomized network at higher edge thresholds (**Fig 5B**) and are therefore providing relevant information regarding the co-expression of transcripts and proteins. It should be noted that this kind of analysis, examining the functional quality of cross-type edges across edge thresholds, is impossible with all other network inference methods tested here as they simply do not create enough cross-type edges (and in many cases do not create any such edges) for analysis.

### Networks derived from permutations of the input data

**Fig 6:**
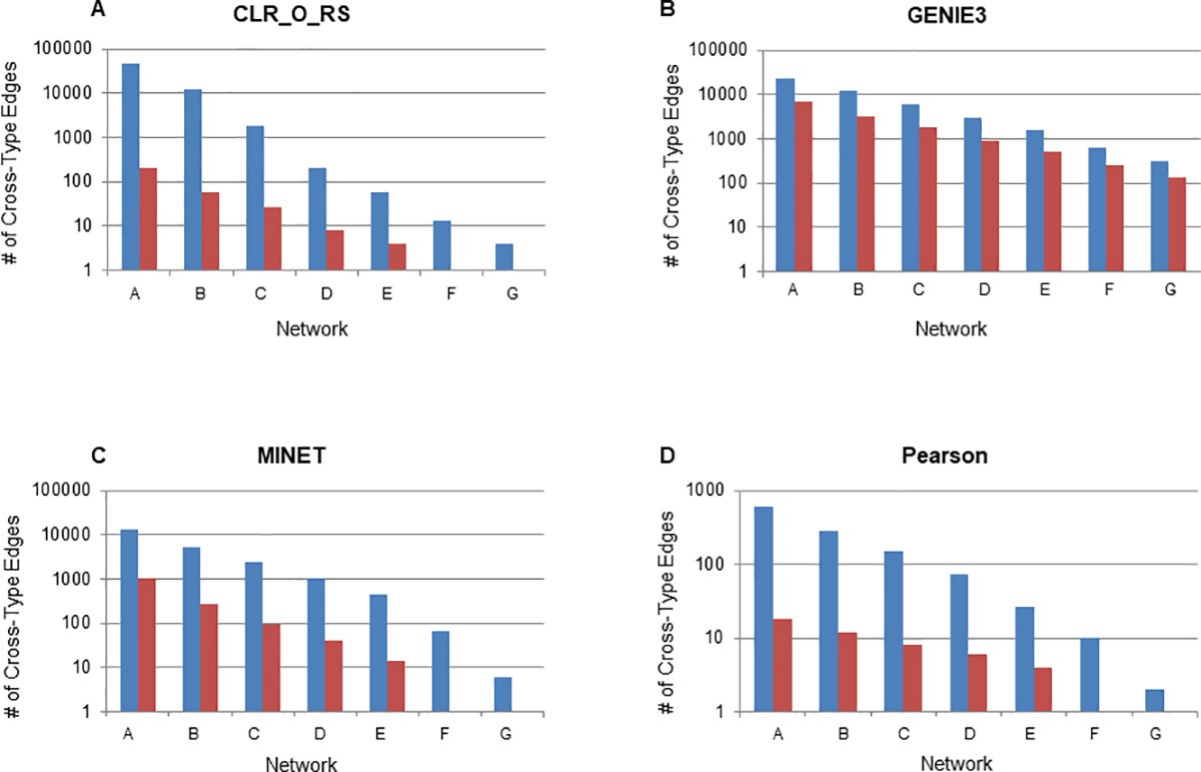
Effects of averaging replicates on cross-type edges. (A) The number of cross-type edges identified in the seven networks using CLR (original algorithm with resampling) when replicates are averaged (blue bars) or kept separate (red bars). (B) Similar analysis with GENIE3, (C) Similar analysis with MINET, (D) Similar analysis with PCC.

Multiple biological factors including post-transcriptional regulation, different rates and mechanisms of RNA and protein degradation, as well as protein modifications contribute to differences in regulation of transcript abundance and protein abundance. However, specific properties of the data arising from the experimental approach used (microarray vs. mass spectrometry), with the distribution of the data, or with the missing values in one type of data also may be contributing to the observed low numbers of cross-type edges. To minimize differences among experimental platforms we next examined the effect of averaging biological replicates within the transcriptomic and proteomic datasets. We explored the effect of averaging in GENIE3 as well as in one representative of each of the three clusters we identified when interrogating network overlap (**Fig 3**), MINET, PCC and the original CLR_O_RS. In all network inference methods and at all edge threshold levels, we saw an increase in cross-type edges when replicates were averaged compared to being kept separate (**Fig 6**). PCC, MINET, and CLR showed the best overall improvement, far better than GENIE3, but this is likely because GENIE3 was already well-suited to drawing cross-type edges and there simply was little room for improvement. However, despite the increase in cross-type edges when replicates were averaged, the functional quality of these edges decreased and, in the case of MINET, CLR_O_RS and GENIE3 networks, was no higher than a randomized network (**S4 Fig**). Averaging replicates also reduces the total number of edges in the network. On average, edge thresholds used to generate networks of the same size as those inferred when replicates were not averaged had to be lowered by 1.6-fold. The loss of input data that is seen when replicates are averaged likely makes it more difficult to draw meaningful edges between features, leading to generally smaller networks if the same edge threshold is used. Therefore, it seems that averaging replicates prior to inferring networks using multi-omic data is not recommended.

**Fig 7:**
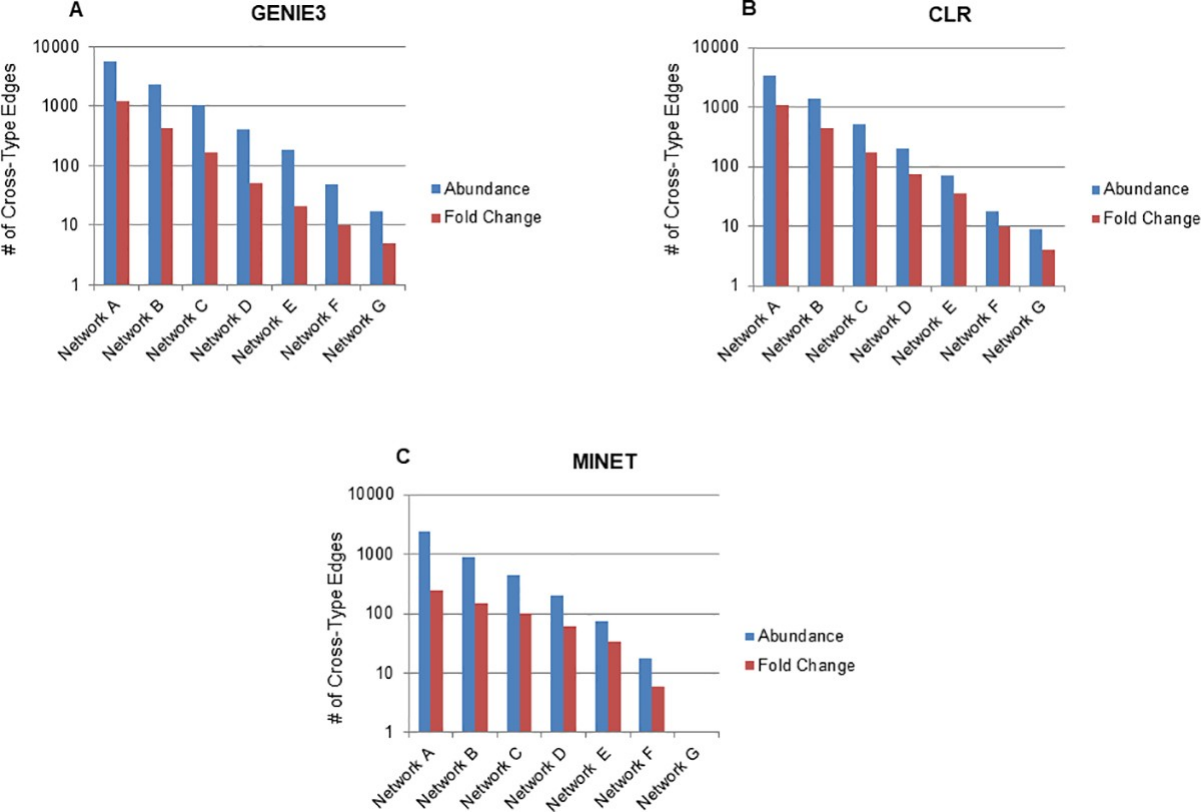
Effects of using fold change values on cross-type edges. (A) Using a smaller dataset of abundance values for proteins and transcripts, networks were inferred using GENIE3 and cross-type edges determined for each network (blue bars). Similar networks were also inferred for fold change values using this same dataset (red bars). (B) The same analysis using CLR instead of GENIE3. (C) The same analysis using MINET instead of GENIE3.

We also tested data that had been normalized to the mean of the row as well as fold change data when comparing to time matched mock infected samples. Normalizing by the mean of the row had no effect on network structure, which is not surprising since most of the inference methods used rely on assessing relative patterns of expression between features that will not be changed by mean normalization. When determining fold change values we normalized all abundance values to uninfected controls and as a result fewer samples were used to infer the network. Mock infected samples were not included as they were used to determine fold change values for infected samples. We also used only proteins with no missing values when determining fold changes across conditions and samples. This smaller dataset contained 38 samples with abundance data for 5000 transcripts and 972 proteins. Because this dataset was different than that used above (it has fewer samples and fewer proteins) we re-inferred networks using abundance values from this smaller dataset as well as fold change values. We tested this dataset using GENIE3 as well as MINET and CLR, as they were the second and third best methods for creating integrated networks from our previous analyses. Interestingly, there was an increase in cross-type edges for both CLR and MINET when using this smaller dataset with abundance values. Despite these increases, GENIE3 was still the best method for inferring cross-type edges in this smaller dataset using abundance values (**Fig 7, S4 Table**). When using fold change values rather than abundance values all three methods inferred far fewer cross type edges. Using fold change data, GENIE3 and CLR were essentially equal in inferring cross-type edges with GENIE3 having slight advantage in very large (200000 edges) and very small (5000–2500 edges) networks and CLR having a slight advantage with medium sized (10000–12500 edges) networks (**Fig 7, S4 Table**). Both CLR and GENIE3 were far better than MINET at inferring cross-type edges using fold change data.

### Ranking of network inference methods using other biological datasets

The above data shows that GENIE3 is the best method for creating integrated networks when using data from *in vitro* cell culture infections. We next wanted to see if this advantage held with other datasets drawn from different experimental setups and with other kinds of–omics data, namely lipidomics. To do so we tested other datasets with GENIE3 as well as MINET and CLR. We first tested another experiment looking at a different strain of Dengue virus (DENV1) infecting the same cell type (U937). This experiment not only included transcriptomic data and proteomic data, as the DENV4 experiment used above did, but also lipidomic data. When looking at edges linking transcripts and proteins in this new dataset GENIE3 was again the highest performer, identifying 81 and 177 cross-type edges in 2500 edge and 5000 edge transcript-protein networks respectively compared to only 1 and 8 edges for CLR and only 1 edge in each network for MINET (**S5 Fig**). As this data included lipidomic data we next looked at cross-type edges in a network comprised of transcripts and lipids, here GENIE3 again performed the best, identifying 15 and 34 cross-type edges in 2500 edge and 5000 edge transcript-lipid networks respectively compared to only 1 and 13 for CLR and 3 and 14 for MINET (**S5 Fig**). Finally, we also looked at a network comprised of proteins and lipids. Here, the advantages of GENIE3 are not as apparent, MINET performs slightly better, though both are better than CLR. GENIE was able to infer 53 and 166 edges compared to 62 and 188 edges for MINET (CLR inferred 2 and 28 edges) (**S5 Fig**). We next examined other datasets that were different from infection of cells with a virus *in vitro*. We examined two datasets looking at *in vivo* infection of mice with influenza virus. With both of these datasets GENIE3 was again, by far, the best method for inferring cross type edges (**S6 Fig**). In addition, we examined both of these datasets specifically looking at the functional edge overlap of cross-type edges. We found, similar to our analysis of Dengue data (**Fig 5B**), that the functional edge overlap of cross-type edges from networks of this influenza data are significantly higher than that seen in a randomized network (**S7 Fig**). The fact that we see this across multiple datasets from very different experiments (human vs. mouse, *in vitro* vs. *in vivo*, various pathogens, etc.) indicates cross-type edges contain biologically relevant data and that this is true across multiple disparate datasets. We also examined another mouse dataset looking at *in vivo* infection with West Nile Virus (WNV). Again, with this dataset GENIE3 was the best at inferring cross-type edges (**S6 Fig**). Finally, we also examined a dataset comprised of transcriptomic and proteomic analysis of ovarian cancer tumors [22]. Here, each tumor represented a unique condition and we chose the 2000 random proteins with the highest variability across tumor samples based on standard deviation. We then combined this dataset with the cognate transcripts matching these proteins for which we had data (a total of 1641 transcripts). With this dataset we found that, across network sizes, there was no significant advantage in using GENIE3, CLR, or MINET regarding cross-type edges. With very large networks (200000 edges) GENIE3 was very similar to MINET and both were slightly superior to CLR. With the smallest network examined (2500 edges) CLR and MINET were slightly superior to GENIE3 with MINET and CLR each inferring about ~500 cross-type edges for this network compared to 347 for GENIE3 (**S6 Fig**). There were also far more cross-type edges inferred with this data set with ~22% of edges linking a protein and a transcript when examining large networks (200000 edges) and ~7% when examining small (5000–2500 edges) networks. While this may have been affected by including a large number of protein-transcript cognate pairs from the same gene, a similar analysis using 2000 random proteins and 2000 random transcripts also found a higher number of cross-type edges, ~20%. This is in contrast to networks examining Dengue infection where only ~3% of the edges were cross-type in a large network inferred with GENIE3 and ~5% were cross-type in a small network.

### Network analysis of antibody-mediated versus receptor-mediated Dengue infection

Having shown with our analysis of Dengue virus infection that GENIE3 is the inference method that is best able to create highly integrated and accurate networks of proteomic and transcriptomic data we applied this approach to comparison of networks derived from receptor-mediated Dengue virus infection and antibody-mediated Dengue virus infection. Studies of DENV3 using multiplicities of infection similar to those used here have shown that Dengue virus can benefit from antibody dependent enhancement during infection. Non-neutralizing monoclonal antibodies against Dengue virus already present in the host can increase invasion of this virus into cells of the immune system including monocytes, dendritic cells and macrophages [46–48]. Infection by the antibody-mediated route can also lead to more serious disease development with increased suppression of host antiviral response [46]. Because of these differences in viral entry into the cell and subsequent changes in disease we examined networks inferred using only data from Dengue infection via an antibody-mediated route and compared that to a network inferred using only data from Dengue infection via a receptor-mediated route. Both transcriptomic and proteomic data was used to infer integrated networks containing 5000 edges using GENIE3, one for antibody-mediated infection data and one for receptor-mediated infection data.

Network centrality in biological networks is an indicator of the importance of the node (transcript or protein) to the function of the network [16, 49, 50]. Pathways with higher centrality in host response networks may be more important to the host response and/or may be more coordinated in terms of their activity. To that end, the centrality of features in KEGG pathways was compared between each network to identify functions that may have higher or lower centrality in an antibody vs. receptor-mediated network. We used betweenness, the number of paths through a network that pass through the gene in question, as a measure of centrality. Studies of centrality in networks of other organisms (including those looking at human pathogens) have shown that betweenness can be used as a proxy for importance or essentiality with genes of higher betweenness being more important [24, 29]. There were a large number of functional categories whose genes showed differences in betweenness between the two networks (**S5 Table**) including those responding to granulocyte-macrophage colony-stimulating factor (GM-CSF) and IL-4. Features that showed decreased expression in response to GM-CSF or IL-4 had, on average, a 2.2-fold higher centrality value in an antibody-mediated network compared to a receptor-mediated network. This difference was significant using a Wilcoxon rank sum test with a p-value of 0.0019 (FDR of 0.144). As with other networks inferred with GENIE3 both the antibody-mediated and receptor-mediated network were highly integrated. Within the category of features showing decreased expression in response to GM-CSF or IL-4 in each network were both proteins and transcripts and several of these had cross-type edges. To further examine the specific contribution from integrated networks made from multi-omic data with cross-type edges (as opposed to networks inferred from only one type of–omic data) we also inferred the same antibody-mediated and receptor-mediated networks but using only transcriptomic data. When comparing these transcriptomic-only networks we were not able to identify any significant difference in the centrality of genes involved in CSF or IL-4. In addition, other pathways showing differences in centrality that were related to viral infection such as response to reovirus infection and cytokine response were also not found in a transcript-only network but only in a network of integrated transcriptomic-proteomic data (**S6 Table**). This observation, combined with the fact that both transcripts and proteins were among those involved with CSF or IL-4 showing changes in centrality, demonstrates the fact that integration of multi-omic data into a network is contributing to the biological results shown here. It is important to note that this in-depth analysis is carried out only on a single dataset and while we do see that cross-type edges contain biological relevant data across other datasets (**S7 Fig**) it will be of interest in future studies to examine these datasets with the level of detail we apply to the Dengue study here. This would reveal whether, and to what degree, cross-type edges can aid in the biological interpretation of networks and what they can show about response of hosts to pathogens or to other stress conditions.

Based on previous analysis of these features they are likely involved in anti-inflammatory pathways as they are downregulated when T cells proliferate upon being stimulated with IL-4 and GM-CSF [51]. As centrality is a measure of importance, the more central position of these features in an antibody-mediated entry network suggests that this route of infection for the virus may lead to downregulation of innate immune pathways and lead to the more enhanced disease outcome seen with antibody-mediated vs. receptor-mediated entry. Other studies have also identified an upregulation of anti-inflammatory cytokines when antibody-mediated entry is undertaken by Dengue [48, 52].

## Discussion

The lack of integration in networks comprised of multiple–omics types can be a serious hindrance to using these networks as accurate, robust and predictive models of biological systems. Using *in vitro* infection data, we show here that GENIE3, a random forest based method, generates, by far, the most integrated networks of ten inference methods tested. This integration does not come at the cost of edge quality as GENIE3 performs as well as other methods at linking features of similar functional roles. Network analysis is a powerful way to view complex systems such as host-pathogen interactions. Using GENIE3, we built integrated networks of data derived from an antibody-mediated Dengue infection experiment and a receptor-mediated Dengue infection experiment and highlight innate immune pathways that are different between networks representing both viral entry mechanisms.

Even when using GENIE3 cross-type edges remain a minority in the *in vitro* dataset we examine, comprising only 3.5–5.4% of total edges in the network. There are a number of reasons why such cross-type edges may not be as prevalent as other edges with the most likely reason being regulatory patterns of the cell that are unique to either proteins or transcripts. However, cell timing of transcription and translation as well as methodological elements of all of network inference approaches examined here may also hinder the formation of cross-type edges. When collecting transcriptomic and proteomic data from a biological system such as an infected cell, transcripts identified as highly expressed or changing their expression patterns act as templates for functional proteins that will be translated at some later time point. This is likely to be more prominent in early time points when systems are still adjusting to the perturbations of the experiment. Because of this time lag, transcripts coding for genes belonging to certain functional pathways will show their expression changes earlier than corresponding proteins of the same pathway. This lag may make it more difficult to identify instances of co-expression between transcripts and proteins of the same functional category. In contrast, transcript pairs belonging to the same functional category and protein pairs belonging to the same functional category, being expressed at roughly the same time, are more likely to have edges. This is likely also the reason why cross-type edges drawn by GENIE3 are not as accurate as edges drawn between two transcripts (**Fig 5B**). It may be of interest to explore methods of building multi-omic networks that attempt to correct this time lag [53]. This might be done by combining early time point transcriptomic data and late time point proteomic data. However, the challenges of combining different -omics datasets from different time points and accurately correcting for the protein translation time lag would be difficult. In addition, we demonstrate here only networks of two omics types (transcript-proteomic, lipid-proteomic, etc.). However, future analyses could infer networks of all three -omics types at once, making a potentially more robust network containing more data.

Despite these challenges and the small number of cross-type edges, GENIE3 does emerge as the best method for inferring integrated networks, specifically of proteomic and transcriptomic data. One of the reasons for this may be the fact that, unlike the other nine methods used, GENIE3 relies on a random forest approach to generate networks. Random forest is an ensemble method that can combine strengths of many weak signals to build better models, and is therefore likely more sensitive to biological signals. It is also able to work with different types of input data and does not require linear relationships between features to establish edges (though this quality is shared by several other methods we examined). Another possible advantage of GENIE3 may lie in its ability to handle values of zero supplied as a substitute for missing values in protein data. When networks were inferred with no missing values for any protein under any condition both CLR and MINET were able to infer more cross-type edges compared to a dataset containing proteins with missing values in some of the conditions. However, GENIE3 was not affected by this difference in the dataset suggesting that this approach may also be especially useful when inferring networks from datasets with missing values, a common feature of proteomic data.

Another observation of interest that emerged from these studies was the functional quality of GENIE3 networks and how this accuracy changes as a function of edge cutoff; smaller, more stringent networks are more accurate than larger networks. This increase in network accuracy among smaller networks was also seen when CLR_O_RS and MINET were used. While all of the networks used here, aside from those that averaged replicates, showed higher accuracy compared to a randomized network, if edge cutoffs are increasingly made less stringent, a point will eventually be reached where the functional edge ratio is no higher than a randomized network. Examining networks made by GENIE3 suggests that this point will be reached with a network of 371535 edges (representing the top 0.77% of all possible edges). The choice of edge threshold is somewhat arbitrary but our analysis suggests that there is a hard lower limit to network size if quality is to be maintained. While making edge threshold less stringent always leads to larger networks, defining a “floor” will help researchers reach a balance between the maximum number of edges and networks of the best quality.

We show here that GENIE3 is the best method for inferring networks from multiple data types, but when networks are built from the same–omics type other methods we tested here may be a better choice. As we described above, MRNETB created networks of the highest functional quality when examining transcript-transcript edges (**Fig 4A**). Other methods also have the advantage of speed with GENIE3 being, by far, the slowest of the 10 methods we tested, taking several hours to run while the other methods, including MRNETB, ran in a matter of minutes. Among the networks tested, only Pearson and Spearman distinguish between positive and negative correlation. Among these two, Spearman was more accurate with a functional edge ratio of 46%, compared to PCC with a functional edge ratio of 36%, suggesting that it may be the superior method when integration is not needed but direction of correlation is. Choice of methods depends on experimental needs; MRNETB is fast and accurate but is poor at integration and does not determine whether nodes are positively or negatively correlated. GENIE3 makes highly integrated networks but is slow and again does not determine whether nodes are positively or negatively correlated. Spearman does determine whether nodes are positively or negatively correlated and is fairly accurate but is very poor at integration. It is also important to point out that the dataset we have here, containing nearly 100 datapoints is actually somewhat small compared to other studies using network inference. While there have been several studies that use datasets of a size similar to ours or smaller [54–56] most studies contain many hundreds of samples. It will be of interest to determine how the choice of network inference method, particularly the use of GENIE3, may be affected by very large sample sizes. It is also likely that autocorrelation may exist in these samples as they are collected from a relatively short time frame. However, since all of the methods are compared across the same datasets if autocorrelation is present it would affect all network inference methods equally.

The advantages of GENIE3 may be less useful when examining multi-omics data from different experimental setups, specifically *in vivo* datasets or multi-omic data where both–omics types are drawn from a mass-spectrometry technology. Here, multi-omics data from short term, *in vitro*, time course experiments led to far fewer cross-type edges compared to clinical data collected directly from patients. There are likely many reasons for this but it may be related to the high amount of sampling during *in vitro* experiments compared to clinical data, with *in vitro* experiments collecting several samples over a matter of hours rather than over weeks or months as is often the case with clinical data. This increased sampling, used with in vitro experiments, may capture cell responses as they are initially responding to stress perturbations with many regulatory signals happening over a short time frame and leading to more discrepancies between cognate transcript-protein pairs. The resulting paucity of cross-type edges amplified the advantage of GENIE3, making this method the only choice for this data type in creating networks with a significant number of cross-type edges. In contrast, data collected from clinical experiments is over a much longer time frame or, in some cases, only a single sample is collected. In this second case regulatory pathways controlling transcript and protein expression may have more time to converge, leading to more correlation in the expression and regulation of cognate transcript-protein pairs, more cross-type edges and the option of using other network inference tools. It may also be that uniform nature and response of cells used in *in vitro* infection experiments may expose and amplify differences between proteins and transcripts. In contrast, samples collected from patients and tumors are a mixture of cell types and responses, which may smooth out inherent differences between transcripts and proteins and lead to more cross-type edges. It is also possible that autocorrelation, which exists in many of the *in vitro* datasets examined here but not the *in vivo* tumor sets may also contribute to the larger number of cross-type edges seen with GENIE3. When examining such clinical data, GENIE3 is no better or worse with regard to cross-type edges than other methods of inferring networks such as CLR and MINET. It should be noted that all three of these methods are still superior to correlation based methods such as Spearman or Pearson. GENIE3 and MINET also find approximately the same number of cross-type edges when examining a proteomic-lipidomic dataset. The fact that a similar result emerges with these two methods only when examining proteomic- lipidomic data, and not proteomic-transcriptomic data or lipidomic-transcriptomic data, suggests that the advantages of GENIE3 may be in linking multi-omic data across different methodological platforms. When one kind of multi-omic data comes from a mass spectrometry platform and one kind comes from a microarray platform (such as proteomic-transcriptomic data or lipidomics-transcriptomic) GENIE3 is by far the best option. However, when both–omics types come from a mass spectrometry platform (such as proteomic-lipidomic) then MINET is able to identify slightly more cross-type edges compared to GENIE3. This suggests that when examining two–omics types that are both mass spectrometry based (proteomics, lipidomics, metabolomics, phospho-proteomics) MINET may be another viable option. It is important to note however that GENIE3 was able to infer a higher functional edge overlap than MINET and thus may be more accurate.

Networks made from multi-omics data are some of the best ways to analyze large datasets and provide a high level but gene and protein-specific view of biological systems. The work presented here provides several approaches for making integrated networks of high functional quality that link different data types. Our approach has also revealed the differing behavior of anti-inflammatory genes in an antibody-mediated versus receptor-mediated network of Dengue infection, information that will be of value as new antiviral treatments are developed for these diseases. While future directions will focus on increasing cross-type edges further and in using network approaches to examine other aspects of viral infections these experiments provide some of the first systematic ranking of methods used to create integrated networks and lay out strategies for how to infer them and which specific methods to use.

## Methods

### Cells and viruses

Viruses were propagated in C6/36 *Aedes albopictus* cells grown in minimal essential medium (Gibco, Grand Island, NY) at 32°C. U937 cells were transfected with a lentivirus vector expressing DC-SIGN, or passaged in parallel, and sorted by FACS. U937 and U937+DC-SIGN cells were maintained in RPMI-1640 (Gibco) at 37°C. Growth media were supplemented with 5% fetal bovine serum (HyClone, Logan, UT), 0.1 mM nonessential amino acids (Gibco), 100 U/ml penicillin and 100 mg/ml streptomycin (Gibco). U937 and U937+DC-SIGN media was supplemented with 2 mM GlutaMAX (Gibco), 10mM HEPES (Cellgro, Manassas, VA). U937+DC-SIGN media included 2-mercaptoethanol (Sigma, St Louis, MO). All infection media contained 2% fetal bovine serum. Cells were incubated in the presence of 5% CO2.

Infectious clones of wild-type strains were constructed using a quadripartite cDNA clone. The DENV4 (Sri Lanka 92) strain was used in the present study. Full-length cDNA was transcribed into genome-length RNAs using T7 polymerase and recombinant viruses isolated in C6/36 cells as previously described. Virus was then passaged twice on C6/36 cells, centrifuged to remove cellular debris, and stored at −80°C as a working stock.

### In vitro experiments

Fc receptor and DC-SIGN mediated entry were titered, and at 24 hours post-infection, conditions were optimized to obtain 60% infection for both entry mechanisms. Each experiment had five infection conditions: U937+DC-SIGN mock infected, U937+DC-SIGN DENV infected, U937 mock infected, U937 DENV+Ab infected and U937 DENV + Ab isotype control (a control condition for Ab-mediated infection). Each condition was examined under four timepoints (2, 8, 16 and 24 hours post-infection). Each timepoint for each condition was examined with five replicates with a few exceptions, four replicates were collected for the following conditions/timepoints: 2 hours post-infection U937+DC-SIGN mock infected, 8 hours post-infection U937+DC-SIGN mock infected, 2 hours post-infection U937 DENV+Ab infected, 24 hours post-infection U937 DENV+Ab infected and 2 hours post-infection U937 DENV + Ab isotype control. With all conditions, timepoints and replicates there were 95 data points comprising this dataset. The virus:antibody/mock mixtures were incubated for 45 minutes in 12-well plates at 37°C. Following this incubation, 1x10^6^ U937 or U937+DC-SIGN cells were added and the infection was allowed to proceed for 2 hours at 37°C. The 2 hour time point was collected, and the rest of the cells were centrifuged for 2 minutes at 450XG and resuspended in fresh infection media. The collection timepoints were 2, 8, 16, and 24 hours post infection. Cells were initially grown and then placed into separate wells for infection assays, with RNA and protein being collected from cells in different wells. For each timepoint cells collected for RNA were pelleted and resuspended in TRIzol. Cells collected for proteomics were filtered, washed twice with sterile PBS, pelleted and dried. Samples were stored at -80°C until processed.

### Transcriptomic analysis

Infected cell samples were pelleted and frozen. Samples were sent to Arraystar (Rockville, MD) for RNA extraction and microarray analysis with the Agilent Human 4 x 44K gene expression array. Total RNA from each sample was quantified using the NanoDrop ND-1000 and RNA integrity was assessed by standard denaturing agarose gel electrophoresis. For microarray analysis, the Agilent Array platform was employed. The sample preparation and microarray hybridization were performed based on the manufacturer’s standard protocols. Briefly, total RNA from each sample was amplified and transcribed into fluorescent cRNA using the manufacturer’s Agilent’s Quick Amp Labeling protocol (version 5.7, Agilent Technologies). The labeled cRNAs were hybridized onto the Whole Human Genome Oligo Microarray (4 x 44K, Agilent Technologies). After washing the slides, the arrays were scanned by the Agilent Scanner G2505C.

### Analysis of transcriptomic data

Background correction was carried out on microarray samples using the maximum likelihood estimation for the normal-exponential convolution model [57], with an offset of 50, as implemented in Bioconductor's [58] *limma* package [59]. Samples were then normalized using quantile normalization so that the entire empirical distribution of each column was identical, this includes log2-transformation of the data. Outliers among samples were detected using intensity distribution and a boxplot graph followed by hierarchical clustering and PCA analysis of expression profiles using the *MVA* package [60]. Any sample that did not visually cluster with other samples of the same condition was removed. For this dataset, only one sample was removed as a result of outlier detection, 1 replicate of the U937 DENV+Ab 24 hour timepoint. To identify differentially expressed probes, we use Bioconductor’s *limma* package [59], which calculates a p-value based on a moderated t-statistic (recommended for experiments with small sample sizes) and then adjusts it to correct for the effects of multiple hypothesis testing. To adjust the p-value, we use the method of Benjamini and Hochberg [61] that controls the false discovery rate (FDR or *q*-value).

### Proteomics sample processing and analysis

Infected cell samples were pelleted and a 2:1 mixture of chloroform:methanol was added to each sample at a ratio of 5:1 over the sample volume, vortexed, and incubated for 5 minutes on ice. Samples were then centrifuged at 12,000 rpm at 4°C for 10 minutes. The upper (aqueous) and lower (organic) layers were removed to fresh tubes, and dried using a Speed-vac. The protein interlayers were washed with ice cold methanol, vortexed, and centrifuged at 12,000 rpm for 10 minutes. Following centrifugation, the methanol was removed from the samples and each was allowed to dry completely. Pellets were then rehydrated in 100 uL of 8 M urea in 50 mM NH_4_HCO_3_ buffer. Protein concentrations were then determined using the bicinchoninic acid (BCA) protein assay (ThermoFisher Pierce, Waltham, MA). Dithiothreitol (DTT, ThermoFisher Pierce, Waltham, MA) was added to each sample to obtain a 5 mM concentration and the samples were incubated at 37°C for 1 hour with shaking at 800 rpm on a Thermomixer (Eppendorf, Hauppauge, NY). Iodoacetamide (ThermoFisher Pierce, Waltham, MA) was added to a final concentration of 40 mM in each sample and then incubated again at 37°C for 1 hour in the dark with shaking at 800 rpm on a Thermomixer. The samples were then diluted 8-fold with 50 mM NH_4_HCO_3_ and CaCl_2_ was added to obtain 1 mM concentration in each sample. Trypsin (1:50 trypsin:protein w:w, USB Affymetrix, Cleveland, OH) was added and the samples were incubated for 3 hours at 37°C with 800 rpm shaking on a Thermomixer. The samples were then flash frozen in liquid nitrogen and stored at -70°C until the solid phase extraction (SPE) cleanup was performed. Samples were thawed, centrifuged at 21,000 x g for 5 minutes at RT and then subjected to C18 SPE cleanup on Strata C18-E 50 mg/1 mL columns (Phenomenex, Torrance, CA) using an automated SPE station (Gilson GX-274, Middleton, WI). Briefly, the columns were conditioned with 3 mL methanol followed by 2 mL of 0.1% trifluoroacetic acid (TFA). After the samples were loaded on the columns, they were rinsed with 4 mL of 95:5 water:acetonitrile with 0.1% TFA. The columns were allowed to dry, after which the samples were eluted with 1 mL of 80:20 acetonitrile:water with 0.1% TFA. The samples were concentrated using a Speed Vac (ThermoFisher Scientific, Waltham, MA) to 50 uL and a final BCA protein assay was performed to quantitate the peptide mass. The samples were diluted to 0.1 ug/uL in water for analysis by LC-MS/MS.

Analysis consisted of reverse-phase LC-MS/MS using Waters nano-Acquity M-Class dual pumping UPLC system (Milford, MA) configured for online trapping and interfaced with a Q-Exactive Plus hybrid quadrupole/Orbitrap mass spectrometer (Thermo Scientific, San Jose, CA). Both trapping and analytical columns were packed in-house using 360 μm o.d. fused silica (Polymicro Technologies Inc., Phoenix, AZ) with 1-cm sol-gel frits for media retention and contained Jupiter C18 media (Phenomenex, Torrence, CA) in 5μm particle size for the trapping column (150 μm i.d. x 4 cm long) and 3 μm particle size for the analytical column (75 μm i.d. x 70 cm long). A 5 μL injection at 5 μL/min was loaded onto trapping column and eluted by reverse direction elution onto the analytical column at 300 nL/min. Mobile phases consisted of (A) 0.1% formic acid in water and (B) 0.1% formic acid in acetonitrile with the following gradient profile (min, %B): 0, 1; 2, 8; 20, 12; 75, 30; 97, 45; 100, 95; 105, 95; 110, 1; 140, 1. The analytical column was coupled to the mass spectrometer with a home built nano-electrospray ionization interface. Electrospray emitters were chemically etched in-house using 150 um o.d. x 20 um i.d. fused silica. The mass spectrometer inlet was maintained at a temperature of 350°C and spray voltage was at 2.2kV. Data were collected for 100 min following a 20 min delay from sample injection. A precursor FT-MS scan was performed from 400–2000 m/z at a resolution of 35k (AGC target 3e6) and the top 12 FT-HCD-MS/MS spectra were acquired in data dependent mode with an isolation window of 2.0 m/z and at a resolution of 17.5k (AGC target 1e5) using a normalized collision energy of 30 and a 30 sec exclusion time.

### Analysis of proteomics data

The initial dataset contained 100 LC-MS instrument runs associated with 100 unique biological samples and 41,947 unique peptides that had at least 2 observations across the 100 biological samples. This corresponded to 5,705 proteins of which 5,699 were human and 6 were viral. The algorithm RMD-PAV [62] was used to identify any outlier biological samples, of which 4 were identified (one replicate each from the control for the receptor-mediated Dengue virus infection at the 2hr and 8hr timepoint, one replicate from the control for the antibody-mediated Dengue virus infection at the 2hr timepoint and one replicate from the antibody-mediated Dengue virus infection at the 2hr timepoint) and confirmed via Pearson correlation. Peptides with inadequate data for either qualitative or quantitative statistical tests were also removed from the dataset [63], resulting in a final dataset ready for normalization that included 96 unique biological samples and 29,694 measured unique peptides corresponding to 4,333 proteins (4,333 human/0 viral). SPANS was used and selected L Order Statistics (LOS) median centering [64] with a parameter of 0.2 for normalization.

Peptides were evaluated with Analysis of Variance (ANOVA) with a Dunnett test correction and a Bonferroni-corrected g-test to compare each virus to the associated mock within each time point. To perform signature-based protein quantification, BP-Quant [65], each peptide was categorized as a vector of length equal to the number of viruses being evaluated. If all comparisons for all time points are 0 for a specific virus it is considered as non-changing and given a value of 0. If there are more time points with an increase in virus to mock than decreasing it is categorized as a +1 and the contrary -1 is given for the decrease in virus to mock. BP-Quant was run with a default parameter of 0.9. All proteins were then analyzed using the same methodology as for the peptides; ANOVA with a Dunnett test correction and a Bonferroni-corrected g-test to compare each virus to the associated mock within each time point.

### Network analysis

For Dengue-derived networks 5000 human transcripts that were differentially regulated (> 2-fold change, *q*-value < 0.05) when comparing virus to time-matched mock samples were selected. We also chose 1930 human proteins that were differentially regulated (> 2-fold change, *q*-value < 0.05) and had missing values in no more than half of the samples examined. Missing values that were present in the protein dataset were replaced with “0” to allow compatibility with all network inference methods. The complete Dengue dataset consisted of 6930 features examined under 20 conditions with either four or five biological replicates queried for each condition, a total of 95 columns of data.

Networks were inferred using 10 feature association metrics that were chosen based on several criteria. We chose to use methods that had been used in the past to infer networks [23, 66–68], we chose methods that used a variety of mathematical approaches to infer networks such as mutual information, correlation, and random forest, and we chose methods that had previously been examined in the DREAM5 challenge based on ranking networks by their ability to link known regulator-target pairs in *Escherichia coli* [42]. The inference methods chosen were Pearson correlation coefficient (PCC), Spearman correlation coefficient, Context Likelihood of Relatedness (CLR), a mutual information based metric [33], six additional mutual information methods in the MINET R package (ARACNE, CLR, MIM, MINET, MRNET, MRNETB) [43], and GENIE3, a random forest method that computes the links between each gene *p* and all other genes *j*^*-p*^ as a function of the predictive nature of *j*^*-p*^ on *p* [69]. Note that the MINET implementation of CLR is somewhat different than the original implementation described by Faith, et. al. [33], so was included here as a related, but distinct, method. The original method uses a binning step to transform continuous expression values in to categorical values before calculation of mutual information and Z-score, the MINET implementation removes this binning step. We also use a resampling approach on the original method [70] that is not used in the MINET implementation. Briefly, this resampling approach consists of inferring several incident networks after randomly removing 20% of the data columns. A consensus network was then made from these incident networks that averaged Z-scores, this consensus network was used for downstream analysis. This resampling with the original CLR algorithm is referred to as CLR_O_RS here to distinguish it from the CLR program used in the MINET package. Transcripts and proteins were preserved as independent nodes in all networks. For each method a threshold was used for each network to define an edge, pairs of features with edge weights above this threshold were included in the network with an edge drawn between them. Once edges were determined to be above the threshold and included in the network all edges were considered equivalent (unweighted). Thresholds used to define edges were chosen for each inference method so that networks of identical size could be compared. Networks ranged in size from 200000 to 2500 edges, representing the top 0.42 and 0.005% of all possible edges respectively (**S4 Table**). For all networks transcripts and proteins were kept as separate nodes in the network, the same gene can be represented by either a transcript or a protein (or both) and each of these is a unique node in the network. We also inferred additional networks using transcriptomic and proteomic data from other studies. These include a publically available dataset of *in vivo* infections of mice with West Nile Virus (WNV) (GEO Accession numbers: GSE77192, GSE77193 and GSE78888), publically available datasets of *in vivo* infections of mice with influenza (GEO Accession numbers GSE68946 and GSE71759) and a dataset examining tumor samples from cancer patients [22].

To compare how well networks agreed with known data and how accurate they were, we compared the ability of networks to draw edges between features within the same functional category. Annotation of features (transcripts and proteins) was obtained from the KEGG database based on the Molecular Signatures Database (MSigDB) [44, 45]. Ratios were determined for edges connecting two annotated features in the same functional category/all edges connecting two annotated features with these ratios expressed as a percentage. This ratio can range from 0% (no edges connecting annotated genes are those connecting genes in the same functional category) to 100% (all edges connecting annotated genes are those connecting genes in the same functional category) and is referred to as the functional edge ratio, a higher functional edge ratio means more edges linked features in the same functional category and the network is more accurate. When randomizing networks for this comparison an identical node-degree distribution was used and only the identity of the individual nodes was shuffled.

## Supporting information

S1 Fig Transcript-protein network made with Pearson correlation coefficient. Using a PCC cutoff of ~0.85 a network of 100000 edges and 5016 nodes was generated. Proteins in the network are colored red and transcripts green. Some small, unconnected clusters of transcripts or proteins have been removed.
(PDF)

S2 Fig Boxplot of transcriptomic and proteomic value distributions. (A) Boxplot showing distributions of transcriptomic and proteomic data. (B) Boxplot showing matched distributions of transcriptomic and proteomic data after multiplying all transcriptomic gene expression values by a factor of 2.6.
(PDF)

S3 Fig Small networks of Pearson correlation, CLR and GENIE3. Networks of 5000 edges were generated using (A) Pearson correlation, (B) CLR and (C) GENIE3. Proteins in the network are colored red and transcripts green. Some small, unconnected clusters of transcripts or proteins have been removed.
(PDF)

S4 Fig Accuracy of network inference methods using averaged replicates. The ratio of cross-type edges connecting annotated features in the same functional category to all cross-type edges connecting annotated features is displayed on the y-axis. The network is displayed on the x-axis. Blue bars represent networks made with GENIE3, red bars represent networks made with CLR (original algorithm with resampling) and green bars represent networks made with MINET. Networks 5, 6 and 7 made using CLR had no cross-type edges with functional annotation and Networks 6 and 7 made using MINET had no cross-type edges with functional annotation so these bars are not displayed.
(PDF)

S5 Fig Comparison of network inference method’s ability to generate cross-type edges in other–omic datatypes. (A) Using lipidomics and proteomics data from infection of human cells with Dengue D1 virus networks were made with three different methods and edge cutoffs were chosen so that all networks of a particular number are the same size across inference methods. The number of Cross-type edges for each network are shown on the y-axis and the methods on the x-axis. (B) A similar analysis as in (A) but looking at proteomic-transcriptomic data from infection of human cells with Dengue D1 virus. (C) A similar analysis as in (A) but looking at lipidomic-transcriptomic data from infection of human cells with Dengue D1 virus.
(PDF)

S6 Fig Comparison of network inference method’s ability to generate cross-type edges in other datasets. (A) Using proteomic and transcriptomic data from infection of mice with Influenza virus (GEO Accession number GSE68946) networks were made with three different methods and edge cutoffs were chosen so that all networks of a particular number are the same size across inference methods. The number of Cross-type edges for each network are shown on the y-axis and the methods on the x-axis. (B) A similar analysis as in (A) but using a different data set examining infection of mice with influenza virus (GEO Accession number GSE71759). (C) A similar analysis as in (A) but using proteomic and transcriptomic data from mice infected with West Nile Virus (WNV). (D) A similar analysis as in (A) but using proteomic and transcriptomic data human ovarian tumor samples.
(PDF)

S7 Fig Functional edge overlaps for mouse influenza networks. (A) Functional edge overlap of cross-type edges in networks inferred from infection of mice with Influenza virus (GEO Accession number GSE68946). Blue bars represent ratios of edges in the networks described here and red bars represent ratios of edges in randomized networks. Error bars indicate standard deviation of ratios from three randomized networks. (B) A similar analysis as in (A) but using a different data set examining infection of mice with influenza virus (GEO Accession number GSE71759).
(PDF)

S1 Table Functional enrichment of transcripts showing differential expression in Dengue virus infected cells vs. mock infected cells at 24 hours post-infection. (XLSX)

S2 Table Functional enrichment of transcripts showing differential expression in antibody-mediated Dengue virus infected cells vs. mock infected cells at 24 hours post-infection. (XLSX)

S3 Table Functional enrichment of proteins showing differential abundance in antibody-mediated Dengue virus infected cells vs. mock infected cells at 24 hours post-infection. (XLSX)

S4 Table Edge cutoffs, network sizes and number of cross type edges for all networks. (XLSX)

S5 Table KEGG functions showing differential centrality in an antibody-mediated network versus a receptor-mediated network built from transcriptomic and proteomic data. (XLSX)

S6 Table KEGG functions showing differential centrality in an antibody-mediated network versus a receptor-mediated network built only from transcriptomic data. (XLSX)
