# Supplementary figures and images for: Unified feature association networks through integration of transcriptomic and proteomic data
Source: PLoS Comput Biol. 2019 Sep 17;15(9):e1007241. doi: 10.1371/journal.pcbi.1007241 (PMC6748406; doi:10.1371/journal.pcbi.1007241)

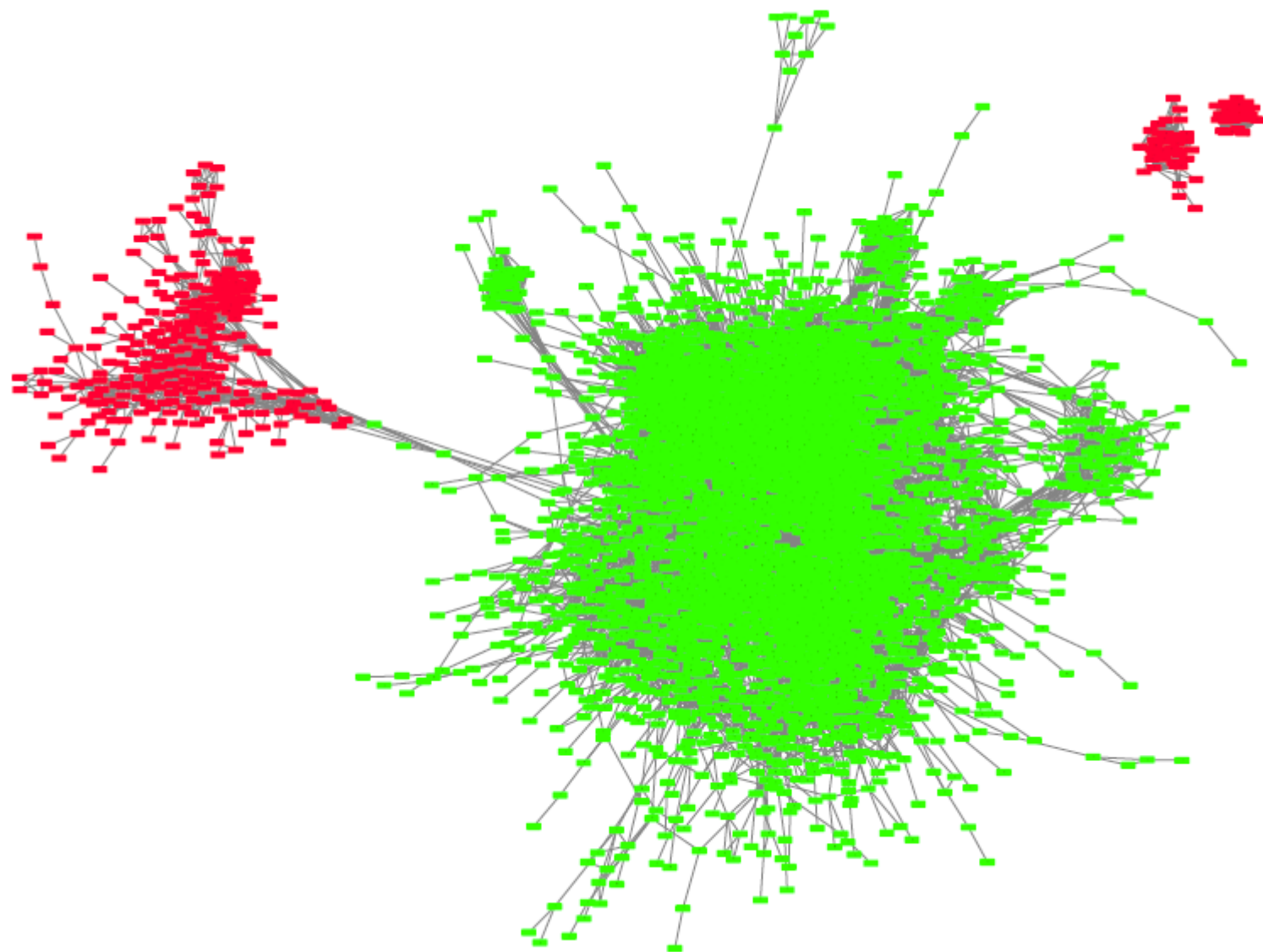

Supplement: S1 Fig — Using a PCC cutoff of ~0.85 a network of 100000 edges and 5016 nodes was generated. Proteins in the network are colored red and transcripts green. Some small, unconnected clusters of transcripts or proteins have been removed. (PDF) [file pcbi.1007241.s001.pdf]

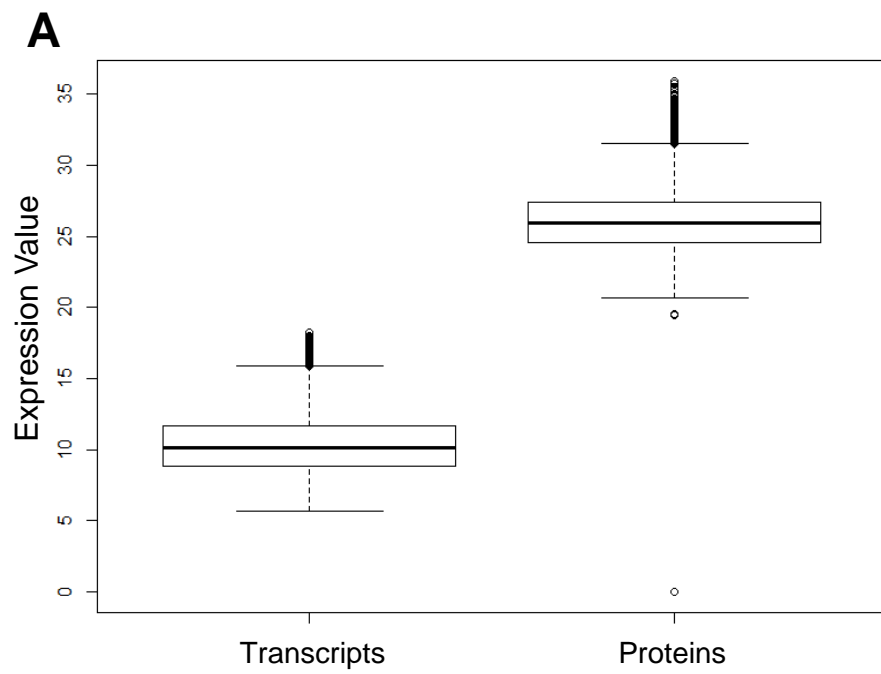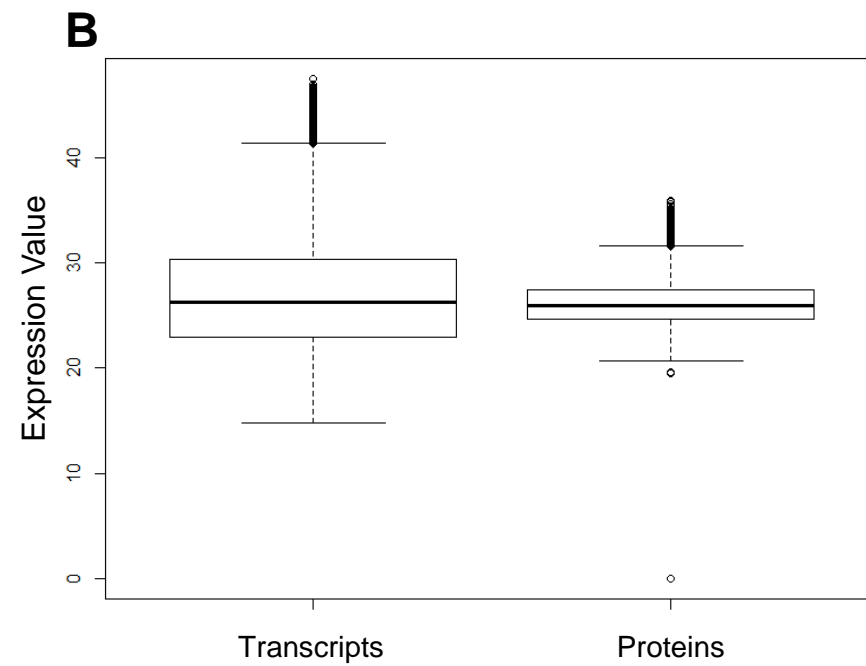

Supplement: S2 Fig — (A) Boxplot showing distributions of transcriptomic and proteomic data. (B) Boxplot showing matched distributions of transcriptomic and proteomic data after multiplying all transcriptomic gene expression values by a factor of 2.6. (PDF) [file pcbi.1007241.s002.pdf]

**A**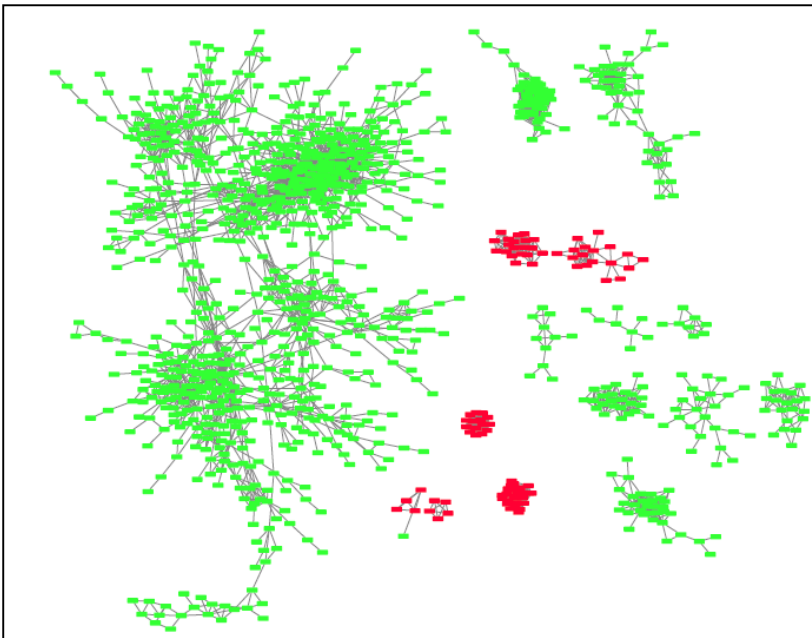**B**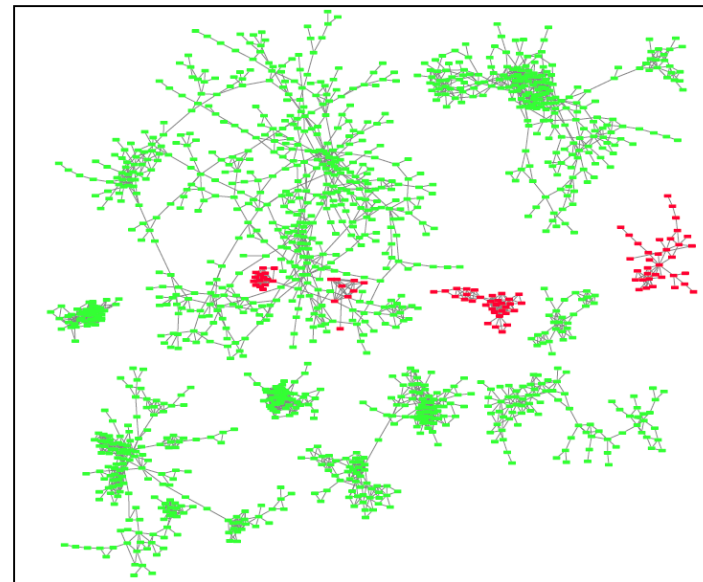**C**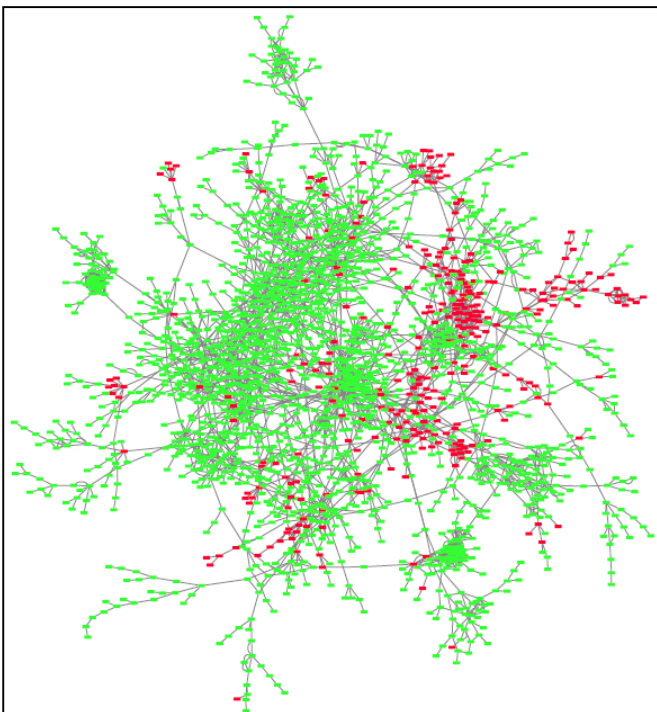

Supplement: S3 Fig — Networks of 5000 edges were generated using (A) Pearson correlation, (B) CLR and (C) GENIE3. Proteins in the network are colored red and transcripts green. Some small, unconnected clusters of transcripts or proteins have been removed. (PDF) [file pcbi.1007241.s003.pdf]

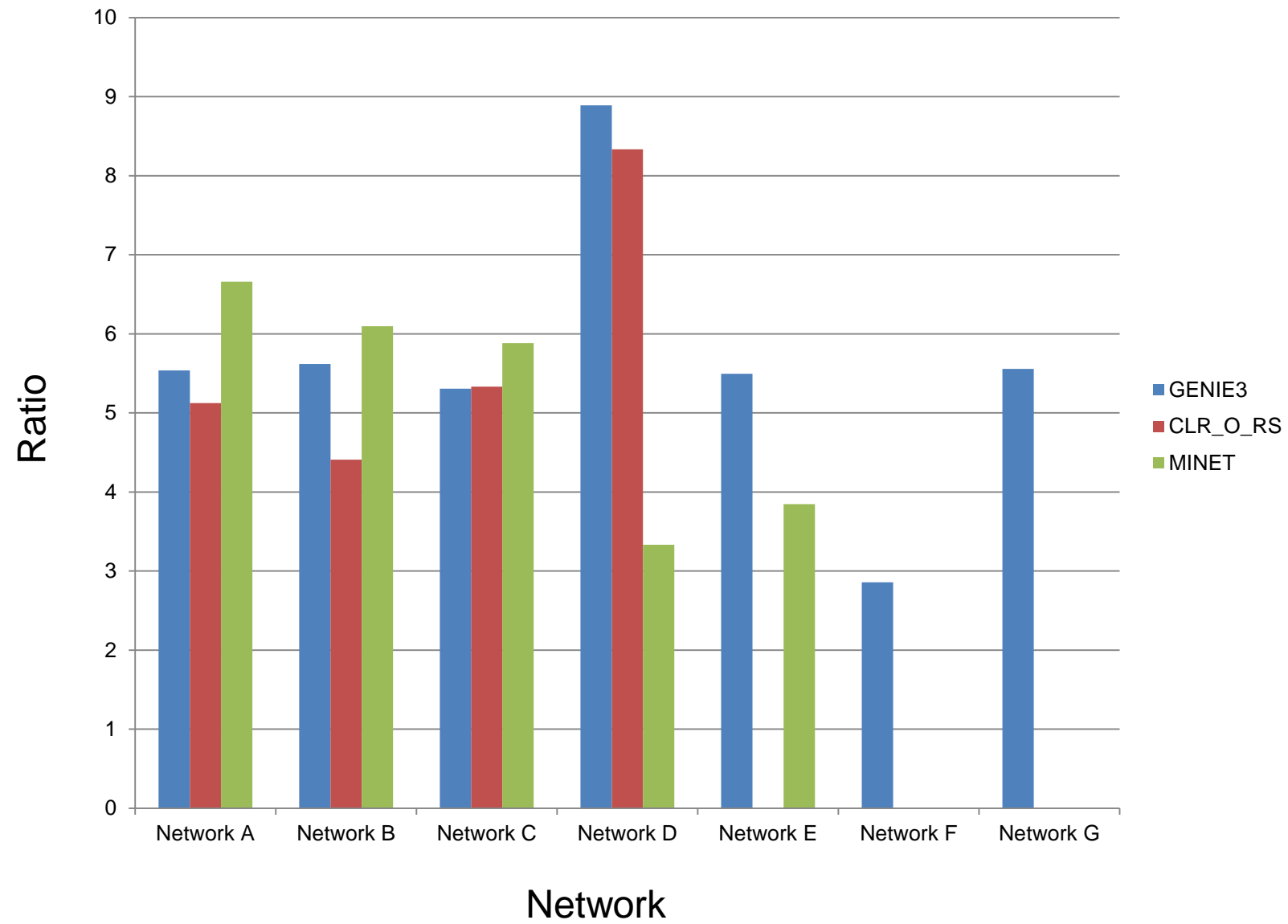

Supplement: S4 Fig — The ratio of cross-type edges connecting annotated features in the same functional category to all cross-type edges connecting annotated features is displayed on the y-axis. The network is displayed on the x-axis. Blue bars represent networks made with GENIE3, red bars represent networks made with CLR (original algorithm with resampling) and green bars represent networks made with MINET. Networks 5, 6 and 7 made using CLR had no cross-type edges with functional annotation and Networks 6 and 7 made using MINET had no cross-type edges with functional annotation so these bars are not displayed. (PDF) [file pcbi.1007241.s004.pdf]

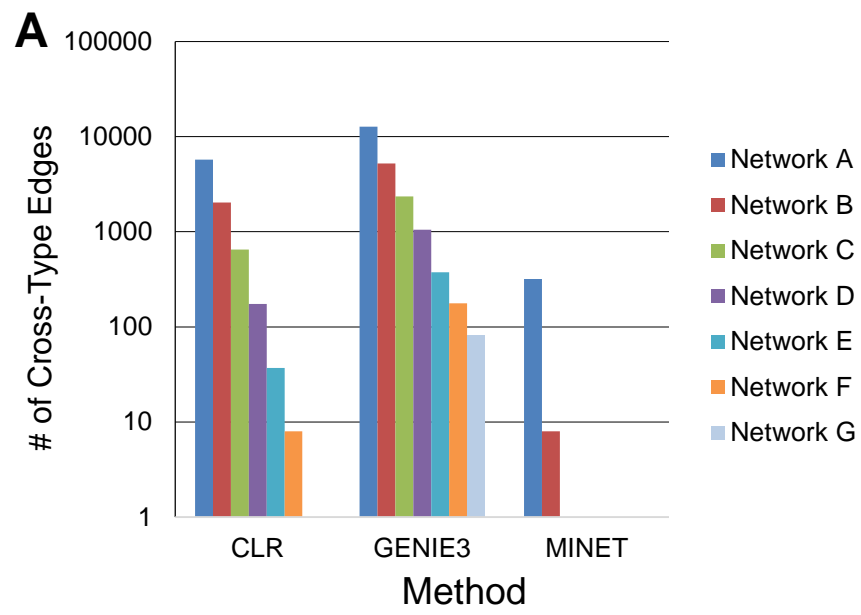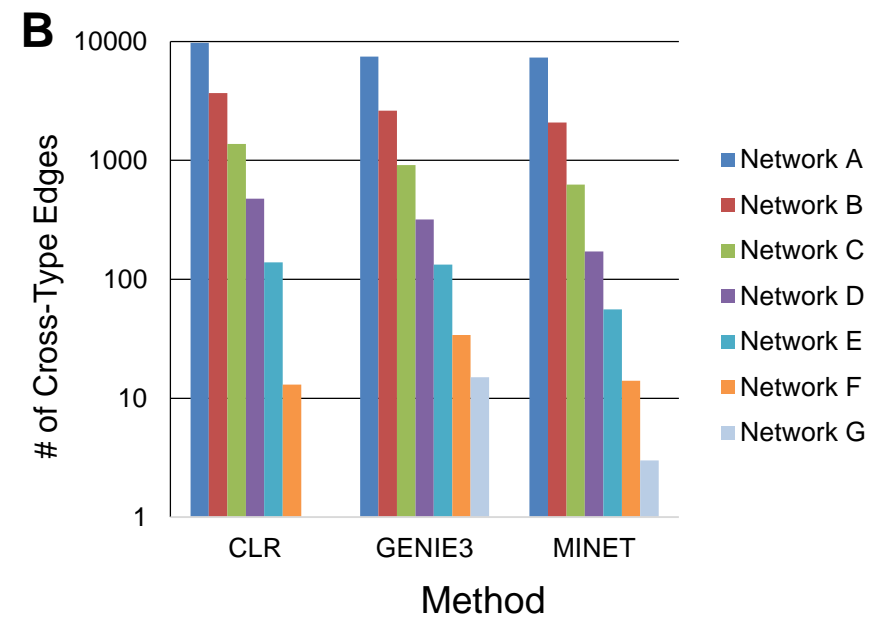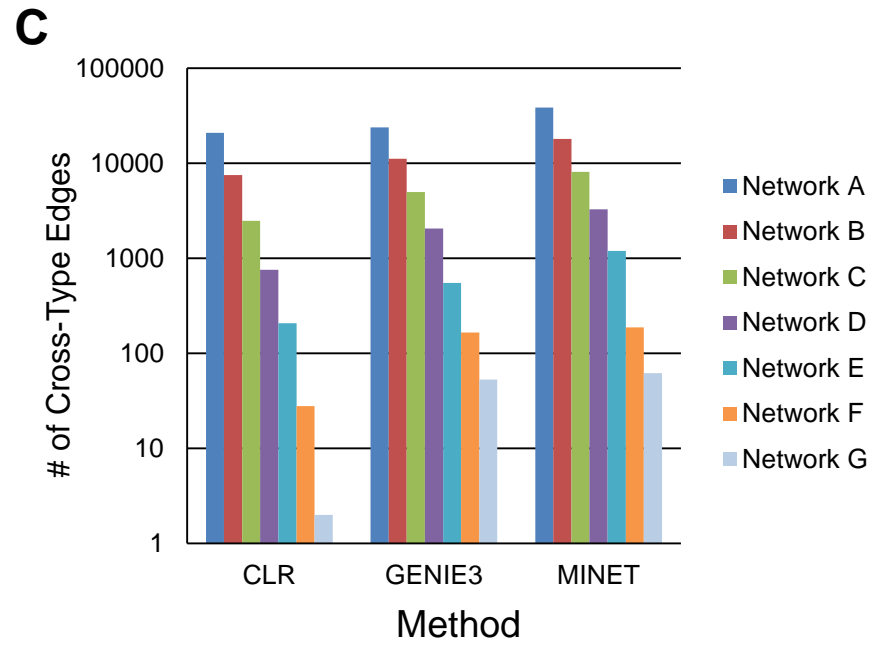

Supplement: S5 Fig — (A) Using lipidomics and proteomics data from infection of human cells with Dengue D1 virus networks were made with three different methods and edge cutoffs were chosen so that all networks of a particular number are the same size across inference methods. The number of Cross-type edges for each network are shown on the y-axis and the methods on the x-axis. (B) A similar analysis as in (A) but looking at proteomic-transcriptomic data from infection of human cells with Dengue D1 virus. (C) A similar analysis as in (A) but looking at lipidomic-transcriptomic data from infection of human cells with Dengue D1 virus. (PDF) [file pcbi.1007241.s005.pdf]

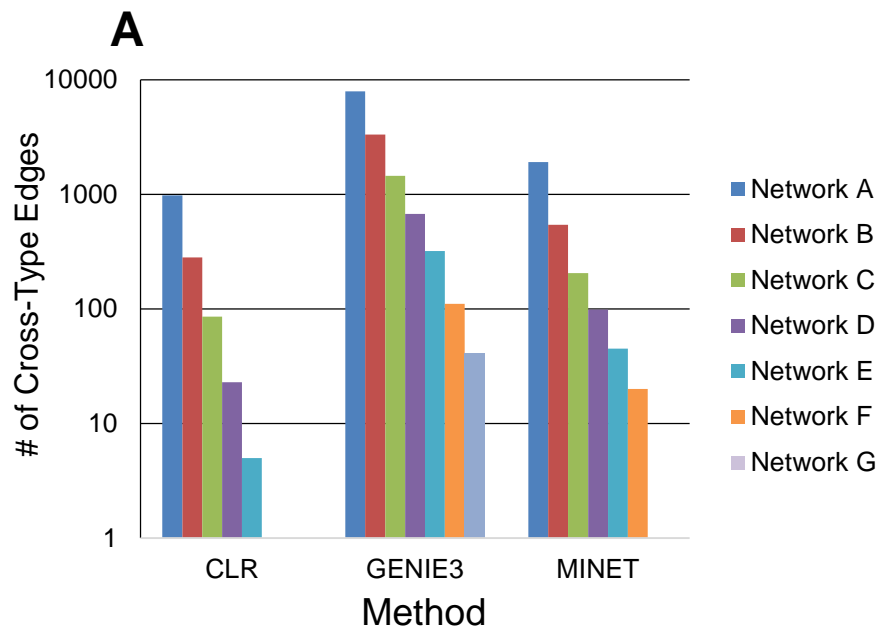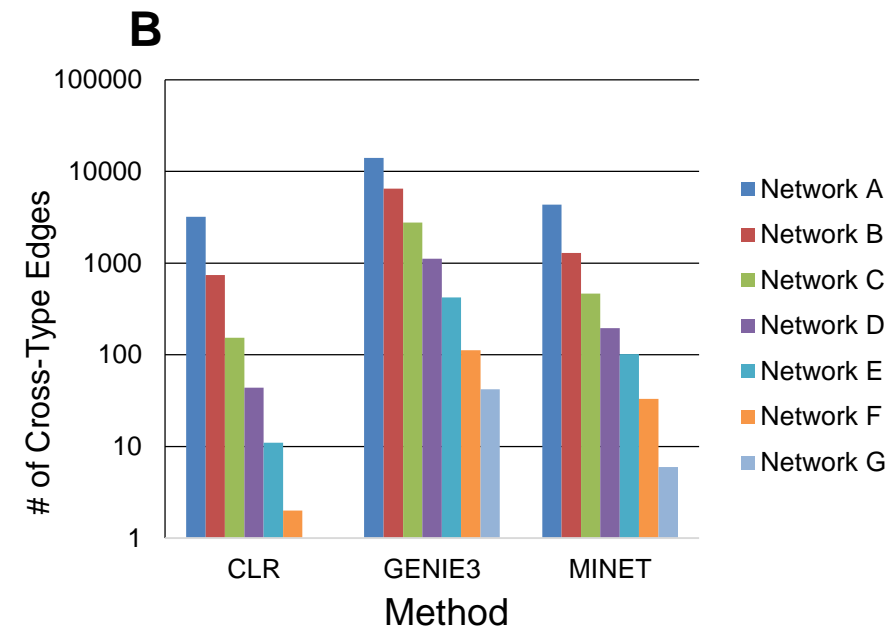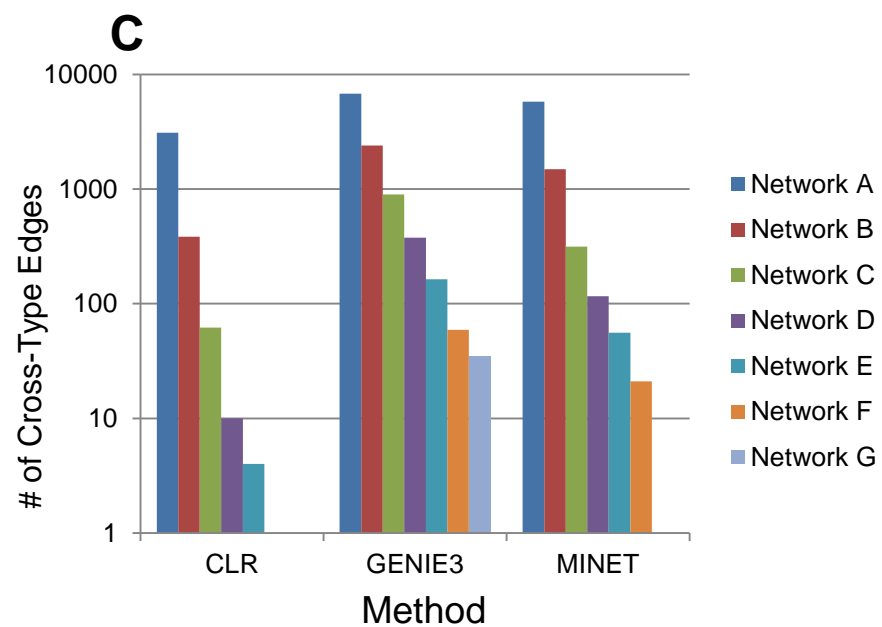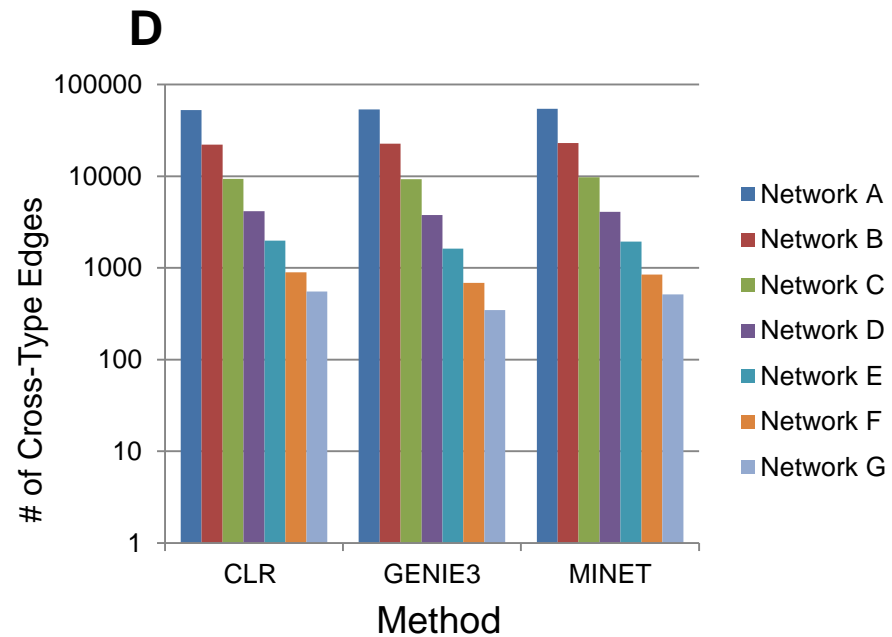

Supplement: S6 Fig — (A) Using proteomic and transcriptomic data from infection of mice with Influenza virus (GEO Accession number GSE68946) networks were made with three different methods and edge cutoffs were chosen so that all networks of a particular number are the same size across inference methods. The number of Cross-type edges for each network are shown on the y-axis and the methods on the x-axis. (B) A similar analysis as in (A) but using a different data set examining infection of mice with influenza virus (GEO Accession number GSE71759). (C) A similar analysis as in (A) but using proteomic and transcriptomic data from mice infected with West Nile Virus (WNV). (D) A similar analysis as in (A) but using proteomic and transcriptomic data human ovarian tumor samples. (PDF) [file pcbi.1007241.s006.pdf]

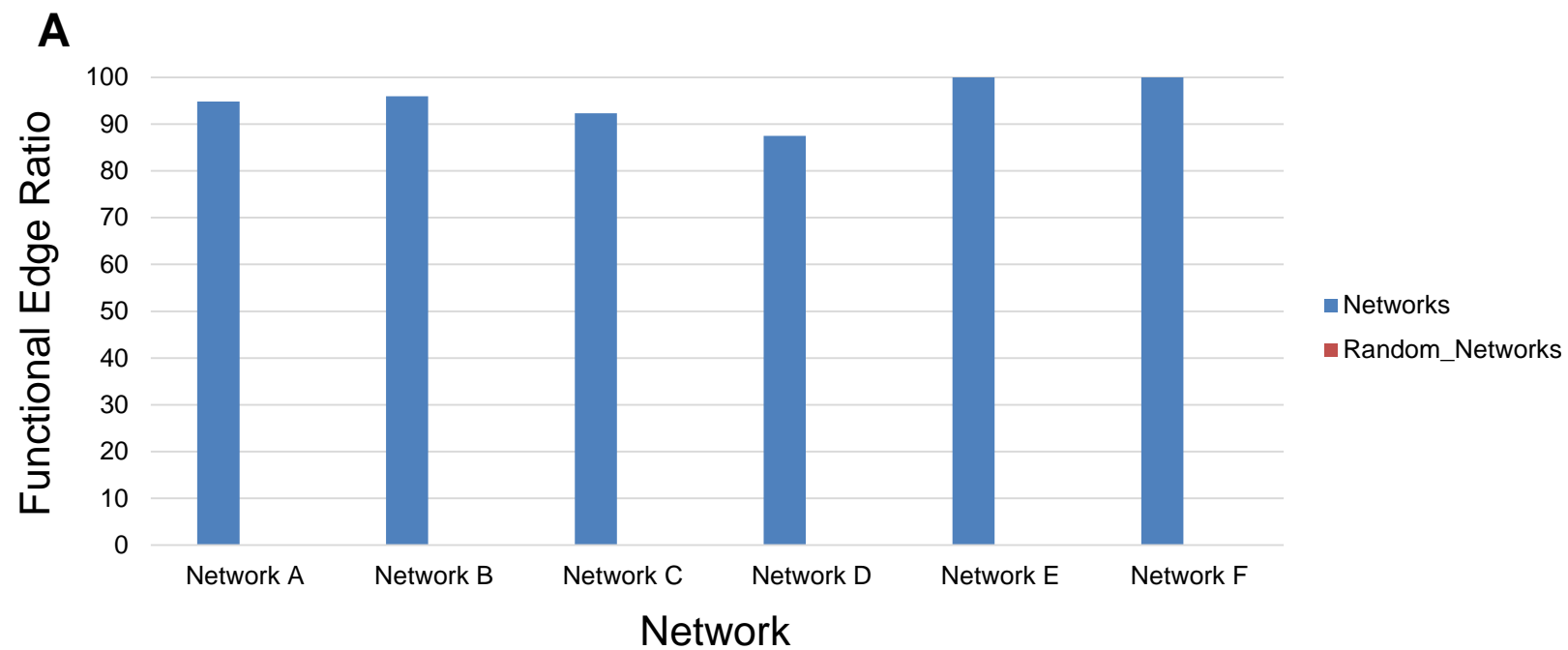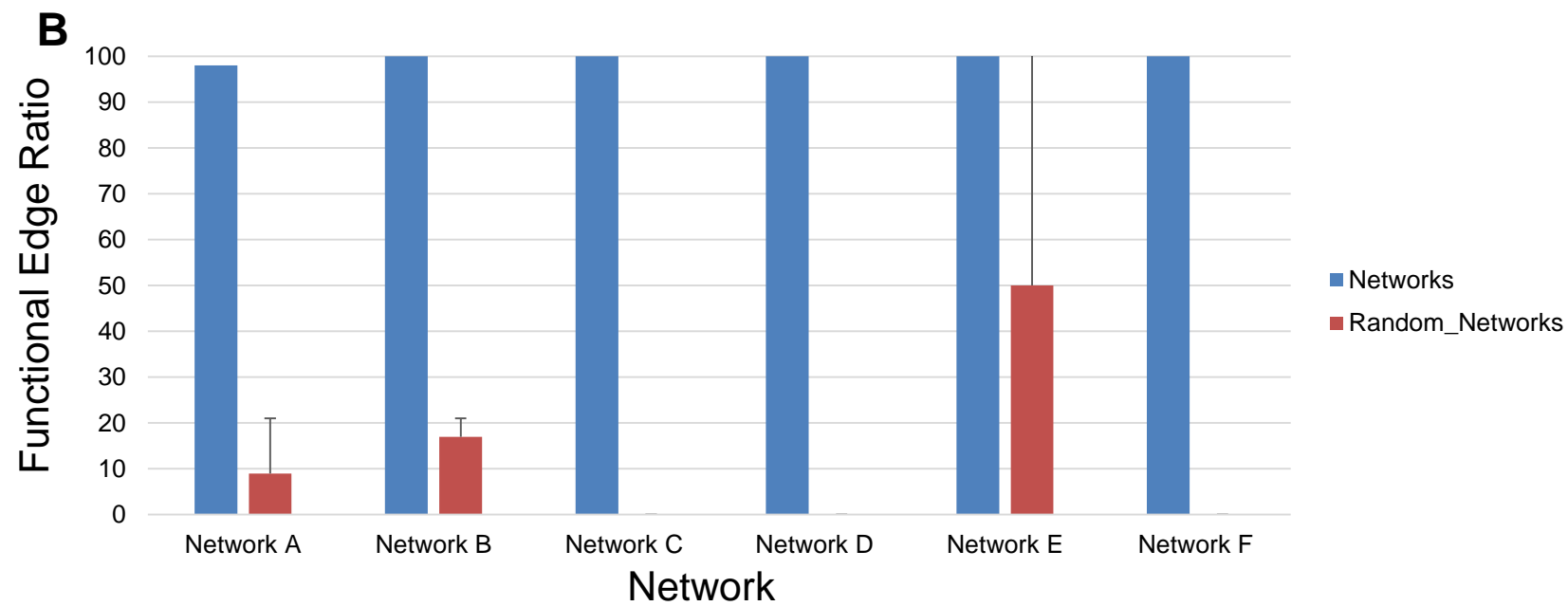

Supplement: S7 Fig — (A) Functional edge overlap of cross-type edges in networks inferred from infection of mice with Influenza virus (GEO Accession number GSE68946). Blue bars represent ratios of edges in the networks described here and red bars represent ratios of edges in randomized networks. Error bars indicate standard deviation of ratios from three randomized networks. (B) A similar analysis as in (A) but using a different data set examining infection of mice with influenza virus (GEO Accession number GSE71759). (PDF) [file pcbi.1007241.s007.pdf]
